# Population Genetic Diversity and Clustering Analysis for Chinese Dongxiang Group With 30 Autosomal InDel Loci Simultaneously Analyzed

**DOI:** 10.3389/fgene.2018.00279

**Published:** 2018-08-02

**Authors:** Bofeng Zhu, Qiong Lan, Yuxin Guo, Tong Xie, Yating Fang, Xiaoye Jin, Wei Cui, Chong Chen, Yongsong Zhou, Xiaogang Li

**Affiliations:** ^1^Key Laboratory of Evidence Science, China University of Political Science and Law, Ministry of Education, Beijing, China; ^2^Department of Forensic Genetics, School of Forensic Medicine, Southern Medical University, Guangzhou, China; ^3^Key Laboratory of Shaanxi Province for Craniofacial Precision Medicine Research, College of Stomatology, Xi’an Jiaotong University, Xi’an, China; ^4^Clinical Research Center of Shaanxi Province for Dental and Maxillofacial Diseases, College of Stomatology, Xi’an Jiaotong University, Xi’an, China; ^5^Department of Anesthesiology, The First Affiliated Hospital of Xi’an Jiaotong University, Xi’an Jiaotong University, Xi’an, China

**Keywords:** InDel, polymorphism analysis, Dongxiang, population genetics, clustering analysis

## Abstract

In comparison with the most preferred genetic marker utilized in forensic science (STR), insertion/deletion analysis possesses further benefits, like absence of stutter peak, low mutation rate, and enabling mixed stain analysis. At present, a total of 169 unrelated healthy Dongxiang individuals dwelling in Dongxiang Autonomous county of Gansu province were recruited in our study to appraise the forensic usefulness of the panel including 30 autosomal diallelic genetic markers. The insertion allele frequencies were in the range of 0.1598 at HLD 111 to 0.8550 at HLD 118. The cumulative match of probability and the combined probability of exclusion were estimated based on independence of pairwise loci, with the values of 3.96 × 10^-11^ and 0.9886, respectively, which showed tremendous potential of this panel to be qualified for forensic personal identification in Chinese Dongxiang group. And it could also be used as a complementary tool for forensic parentage testing when combined with standard STR genetic markers. Furthermore, calculation of the *D_A_* distance and *F_st_* values of pairwise populations, phylogenetic reconstruction, multidimensional scaling analysis, structure clustering analysis were also conducted to probe the genetic relationships between Dongxiang group and the other 30 reference populations. Results demonstrated that Dongxiang ethnic group might be genetically closer related with most Chinese populations involved in our study, especially Tibet groups, Xibe group, and several Han populations.

## Introduction

Insertion/deletion (InDel) polymorphic genetic marker characterized by abundance in the genome, relative low mutation rate, small amplicon size, and compatibility with current genotyping platform (Wei et al., 2014) is gradually becoming a possible alternative approach for forensic amplifications to overcome some inevitable limitations of traditional STRs, such as stutter products, high mutation rate, and so on. In recent years, it has been proved that the InDels could be useful in human identification (Pereira et al., 2009), mixed stain identification and deconvolution (Oldoni et al., 2017), as well as population genetic analysis including biogeographic ancestry inference and population substructure determination (Santos et al., 2010). Sun et al. (2016) verified the considerable potency of a multi-InDel panel in ancestry inference of subpopulations in China and Caputo et al. (2017) reported the potential use of a 33 X-InDel panel in Argentina populations. A year later, Hwa et al. (2018) also reported a panel’s efficiency in degraded and non-degraded DNA mixtures with SNPs and InDels simultaneous analyzed via MPS technology. The commercially available Investigator DIPplex kit which contains 30 autosomal InDel loci and an amelogenin gene has been testified in a large majority of populations to evaluate its efficiency for forensic applications. Chinese population data including Tujia (Shen et al., 2016), Uyghur (Mei et al., 2016), Xibe (Meng et al., 2015), Hui (Xie et al., 2018), Dong (Li et al., 2015), Tibetan (Guo et al., 2016), Kazak (Kong et al., 2017), Zhuang (Li et al., 2015), and Yi (Li et al., 2015) were reported previously. But data in existence did not incorporate Dongxiang ethnic minority in Gansu province of China. And that’s the reason why we chose Dongxiang group as our research subject.

As a country with a long-civilized history, China is also universally accepted to be one big oriental country composed of 56 ethnics and full of modern vitality. Diversities of ethnics and cultures make China a tremendous treasure to conduct genetic-related analysis. Dongxiang group is one of the Muslin ethnic groups mainly distributed in Gansu province and Xinjiang Uyghur Autonomous Region, China. According to the 2010 census, the population of Dongxiang reach 515,000 (Wen et al., 2013). Making a comparison between the population size of Dongxiang and the other ethnic minorities of China explains the constriction of our sample size. On account of lacking convincing historical records, the origin of Dongxiang group is not explicit and continuously debated by historians until now. But researchers have exerted a certain amount of studies concentrating on this issue based on diverse genetic markers, like Y-STRs (Wang et al., 2003), mitochondria DNA (Zhang et al., 2015), and so on. In present study, a panel of 30-InDel loci was firstly applied to Dongxiang group with the cumulative match of probability (CMP) and cumulative probability of exclusion (CPE) calculated to assess its forensic efficiency in this region. Besides, phylogenetic reconstructions, multidimensional scaling analysis (MDS), structure clustering analysis, heatmaps of fixation index (*F_st_*), and *D_A_* values of pairwise populations were constructed based on these 30 InDels to explore the interpopulation genetic relationships between Dongxiang and 30 reference populations.

## Materials and Methods

### Sample Collection and Ethics Statement

A total of 169 healthy unrelated Dongxiang individuals were recruited in our study from Dongxiang Autonomous county of Gansu province. All the individuals declared on kinships among them within at least three generations and no immigration events happened in their family history. Not until the written informed consents were acquired from each of them did we further continue our research. Five milliliters peripheral blood was collected and the genomic DNA was extracted by paramagnetic particle method according to the manufacturer’s recommendation. Procedures involved in our experiment were in good agreement with the human and ethical research principles of Southern Medical University and Xi’an Jiaotong University, China. Genotypic data of the 30-InDel loci for 169 Dongxiang individuals could be found in the public database named “figshare” (10.6084/m9.figshare.6743057).

### PCR Amplification and Subsequent InDel Genotyping

Thirty InDel loci were co-amplified in a single PCR system with the necessary reagents and reaction conditions strictly set following the manufacturer’s protocol of Investigator DIPplex commercial kit (Qiagen, Hilden, Germany) in GeneAmp PCR 9700 Thermal Cycler (Applied Biosystem, Foster City, CA, United States). Subsequent genotyping of PCR products was performed in an ABI 3500XL Genetic Analyzer (Applied Biosystem, Foster City, CA, United States) according to the manufacturer’s recommendation and alleles allocation were operated by GeneMapper *ID-X* version 1.5 software (Applied Biosystem, Foster City, CA, United States). Positive control as well as negative control was also included to ensure precise results of InDel genotyping.

### Statistical Analysis

Calculations of the insert/deletion allele frequencies and forensic statistical parameters incorporating match probability (MP), discrimination power (DP), probability of exclusion (PE), polymorphism information content (PIC), and observed heterozygosity (Ho) of the 30 InDels were implemented by modified PowerState version 1.2 spreadsheet. Linkage disequilibrium (LD) analysis was carried out by SNPAnalyzer version 2.0 (ISTECH, Goyang, South Korea) software (Yoo et al., 2008). Locus-by-locus *P*-values for interpopulation differentiation comparisons were conducted in Arlequin version 3.5.1.2 software (Excoffier et al., 2007). Expect heterozygosity was calculated by DISPAN program. A MDS plot at population level was performed to reveal the spatial clustering status of studied Dongxiang group and the 30 reference populations with SPSS 20.0 software. *D_A_* distance values and *F_st_* values were calculated by DISPAN program^1^ and Genepop version 4.0 software (Rousset, 2008), respectively. Subsequently, a collection of heatmaps of deletion allele frequencies of the 30-InDel loci, *D_A_* distance values and *F_st_* values of pairwise populations were performed with pheatmap package by *R* version 3.4.5 statistical software. A phylogenetic tree was conducted based on *D_A_* distance values by employing neighbor-joining method with MEGA version 6.06 software^2^. And an unrooted tree was also generated based on allele frequencies of 30-InDel loci by Phylip version 3.69 software^3^. Clustering structure analysis was performed via STRUCTURE version 2.3.4 software.

## Results and Discussion

### Hardy–Weinberg Equilibrium (HWE) Tests for 30 Loci and Pairwise Loci Linkage Disequilibrium (LD) Analysis

With significance level for probability values set at 0.00167 (*P* = 0.05/30) after Bonferroni’s correction (Goeman and Solari, 2014) for multiple tests, no remarkable deviation from Hardy–Weinberg equilibrium (HWE) was observed for the 30 InDels in Dongxiang group. And locus-specific *P*-values were represented in **Table 1**. In addition, LD tests of pairwise InDel loci were also carried out to assess the independence of each InDel locus. As shown in **Supplementary Figure S1**, 435 small blocks represented 435 kinds of interclass correlation tests for 30 InDels. None of the blocks was covered by crimson and no area was encircled by thick black lines with the *r*^2^ threshold established at 0.8 level, revealing no LD existed between any of two different InDel loci. More detailed information about several indices for LD was presented in **Supplementary Table S1**. In combination with the results of HWE tests and LD analysis, we concluded that our population data were representative and the 30-InDel loci were independent of each other. Thus, the product law could be unquestionably utilized to calculate the cumulative match of probability (CMP) and cumulative probability of exclusion (CPE).

**Table 1:** Allele frequencies and forensic efficiency parameters of the 30-InDel loci for Chinese Dongxiang group (*n* = 169).

HLD	rs#	Allele frequency(insertion/deletion)	MP	DP	PIC	PE	TPI	He	*P*
HLD 77	1611048	0.4586/0.5414	0.3481	0.6519	0.3733	0.1266	0.8622	0.4980	0.0428
HLD 45	2307959	0.6746/0.3245	0.4072	0.5928	0.3427	0.1306	0.8711	0.4404	0.7075
HLD 131	1611001	0.4320/0.5680	0.3672	0.6328	0.3703	0.1560	0.9286	0.4922	0.4253
HLD 70	2307652	0.5680/0.4320	0.3828	0.6172	0.3703	0.1849	0.9941	0.4922	0.8997
HLD 6	1610905	0.4911/0.5089	0.3681	0.6319	0.3749	0.1749	0.9713	0.5013	0.6752
HLD 111	1305047	0.1598/0.8402	0.5705	0.4295	0.2324	0.0527	0.6870	0.2693	0.9319
HLD 58	1610937	0.3905/0.6095	0.3737	0.6263	0.3627	0.1387	0.8895	0.4774	0.3030
HLD 56	2308292	0.5799/0.4201	0.3708	0.6292	0.3685	0.1560	0.9286	0.4887	0.4802
HLD 118	16438	0.8550/0.1450	0.6140	0.3860	0.2172	0.0285	0.6213	0.2486	0.1084
HLD 92	17174476	0.3669/0.6331	0.3852	0.6148	0.3566	0.1387	0.8895	0.4659	0.4646
HLD 93	2307570	0.5473/0.4527	0.3939	0.6061	0.3727	0.2119	1.0563	0.4970	0.4409
HLD 99	2308163	0.8462/0.1538	0.5766	0.4234	0.2265	0.0572	0.6983	0.2611	0.4980
HLD 88	8190570	0.5266/0.4734	0.3750	0.6250	0.3743	0.1849	0.9941	0.5001	0.9374
HLD 101	2307433	0.4734/0.5266	0.3693	0.6307	0.3743	0.1749	0.9713	0.5001	0.6993
HLD 67	1305056	0.5651/0.4349	0.3738	0.6262	0.3707	0.1700	0.9602	0.4930	0.7218
HLD 83	2308072	0.3905/0.6095	0.3820	0.6180	0.3627	0.1560	0.9286	0.4774	0.6789
HLD 114	2307581	0.3077/0.6923	0.4419	0.5581	0.3353	0.1749	0.9713	0.4273	0.1281
HLD 48	28369942	0.3994/0.6006	0.3760	0.6240	0.3647	0.1515	0.9185	0.4812	0.5059
HLD 124	6481	0.5947/0.4053	0.4073	0.5927	0.3659	0.2119	1.0563	0.4835	0.2619
HLD 122	8178524	0.3254/0.6746	0.4041	0.5959	0.3427	0.1228	0.8535	0.4404	0.4933
HLD 125	16388	0.4527/0.5473	0.4334	0.5666	0.3727	0.2742	1.2071	0.4970	0.0209
HLD 64	1610935	0.7988/0.2012	0.5119	0.4881	0.2698	0.0773	0.7478	0.3224	0.8026
HLD 81	17879936	0.7633/0.2367	0.4723	0.5277	0.2961	0.0719	0.7348	0.3624	0.2462
HLD 136	16363	0.5148/0.4852	0.3864	0.6136	0.3748	0.2063	1.0432	0.5010	0.6091
HLD 133	2067235	0.3432/0.6568	0.4420	0.5580	0.3492	0.2176	1.0696	0.4522	0.0358
HLD 97	17238892	0.3669/0.6331	0.3982	0.6018	0.3566	0.1652	0.9494	0.4659	0.8462
HLD 40	2307924	0.6272/0.3728	0.4003	0.5997	0.3583	0.1749	0.9713	0.4690	0.6732
HLD 128	17878444	0.3728/0.6272	0.3860	0.6140	0.3583	0.1472	0.9086	0.4690	0.6149
HLD 39	2307956	0.2041/0.7959	0.5095	0.4905	0.2721	0.0919	0.7824	0.3259	0.3310
HLD 84	3081400	0.6746/0.3254	0.4189	0.5811	0.3427	0.1560	0.9286	0.4404	0.5792


*MP, match of probability; DP, discrimination power; PIC, polymorphic information content; PE, probability of exclusion; TPI, typical paternity index; He, expected heterozygosity; P, probability values for Hardy–Weinberg equilibrium tests.*

### Allele Frequency Diversities and Forensic Efficiency Parameters

To further evaluate the forensic potency of the 30-InDel panel applied in Dongxiang ethnic group, InDel allele frequencies as well as forensic efficiency parameters of the 30 InDels were also calculated and the results were presented in **Table 1**. The insertion allele frequencies were in the range of 0.1598 at HLD 111 to 0.8550 at HLD 118, with 93% of the InDel markers over 0.3. The MP, DP, PE, PIC, and He values were in the range of 0.3481 at HLD 77 to 0.6140 at HLD 118; 0.3860 at HLD 118 to 0.6519 at HLD 77; 0.0285 at HLD 118 to 0.2742 at HLD 125; 0.2172 at HLD 118 to 0.3749 at HLD 6; 0.2486 at HLD 118 to 0.5013 at HLD 6, respectively. The CMP and CPE were calculated with a final value of 3.96 × 10^-11^ and 0.9886, respectively. Hence, we verified the 30 diallelic InDel panel can be served as an effective tool for personal identification as well as a supplement to paternity testing for forensic applications in Chinese Dongxiang group.

### Interpopulation Genetic Diversity Analysis

As displayed in **Supplementary Table S2**, locus-by-locus *P*-values of interpopulation genetic differentiations between Dongxiang group and the 30 reference populations were calculated on the basis of allele genotyping data of the 30-InDel loci with the AMOVA method utilized. Significance level of *P*-value was adjusted to 0.0017 (*P* = 0.05/30) with the Bonferroni’s correction applied, and no significant differences were observed between Dongxiang and Yi group in Sichuan at all the 30 loci, one locus differentiation with Tibet Tibetan, Qinghai Tibetan, Beijing Han, and Chengdu Han populations, three loci difference with Uyghur, Xibe, Miao, and Tujia, Zhuang groups, four loci differentiation with Hui, Kazak, She groups, and Henan Han population, five loci differentiation with Guangdong Han population, and six loci difference with Shanghai Han population. By contrast, significant differentiation was observed between Dongxiang group and six Mexican populations (Jalisco Mexican, Veracruz Mexican, Chihuahua Mexican, Mexico Mexican, Yucatan Mexican, and Amerindian Mexican ) at 6, 7, 8, 11, 12, and 12 loci, as well as four European populations [Central Spanish (Martín et al., 2013), Basque (Martín et al., 2013), Dane (Friis et al., 2012), and Hungarian (Kis et al., 2012)] at 10, 11, 12, and 14 loci. And two African indigenous populations [Zulu (Hefke et al., 2016) and Xhosa (Hefke et al., 2016)] were detected to be most significantly different from Dongxiang group at 21 and 22 loci, respectively. Clearly, compared with non-Chinese populations, closer genetic relationships might be existed between Dongxiang and the other Chinese populations. As for single locus diversities, the first four loci shown greatest remarkable diversities between Dongxiang and the reference populations were HLD 118, HLD 39, HLD 111, and HLD 99, of which HLD 118 and HLD 99 displayed differentiations among all the non-East Asian populations.

### A Heatmap of Deletion Allele Frequency Distributions of the 30-InDel Loci for 31 Populations

**FIGURE 1:**
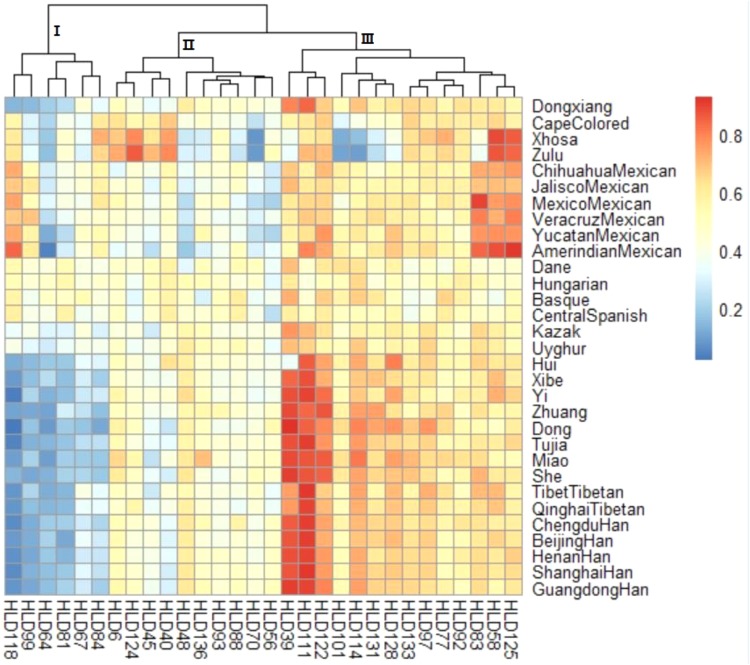
A heatmap of deletion allele frequency distributions for Chinese Dongxiang group and the 30 reference populations. Blue for the lowest and red for the highest deletion allele frequency.

Additionally, a heatmap of deletion allele frequencies for the 30 loci was also performed. As shown in **Figure 1**, the color of each block deepened with the corresponding deletion frequencies increasing. The color scale ranged from blue for the lowest deletion allele frequency to red for the highest deletion allele frequency. And clustering analysis for the 30-InDel loci was also generated on the top of the figure and three primary clusters were easily distinguished. It was clear that cluster 1 (HLD 118, HLD 99, HLD 64, HLD 81, HLD 67, and HLD 84) exhibited relative small deletion allele frequencies in most Chinese populations in exception of Kazak and Uyghur groups while a small branch of cluster 3 (HLD 39, HLD 111, and HLD 122) showed larger deletion allele frequencies in these populations. Hence, we speculated these above-mentioned loci might be potential for biogeographic ancestry inferences for Chinese populations involved in our study. On the part of distributions of deletion allele frequencies for the 30 loci, Dongxiang group was discovered to share analogical deletion allele frequency distributions with several Chinese populations (Qinghai Tibetan, Tibet Tibetan, Chengdu Han, Beijing Han, and Henan Han) while distinct deletion allele frequencies distributions with most non-Chinese populations, which meant a similar genetic structure among Dongxiang and these Chinese populations. Furthermore, observations showed the deletion allele frequencies of the 30 loci were approximately identical among the only four European populations included in our study, which indicated the 30-InDel panel could be suitable for personal identification cases in these populations.

### *D_A_* and *F_st_* Values of Pairwise Populations

**FIGURE 2:**
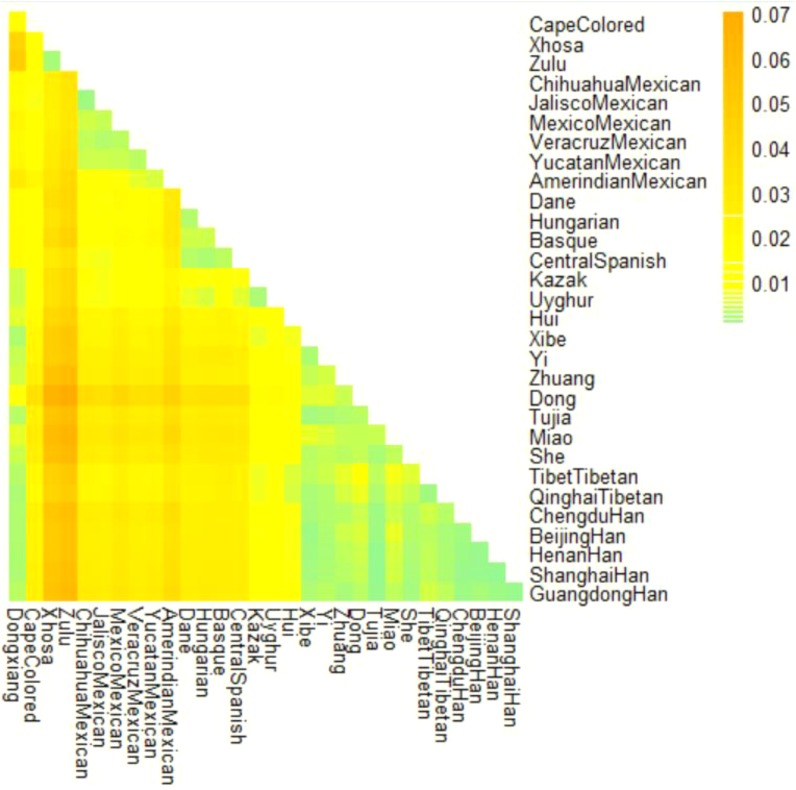
A heatmap of *D_A_* distance values of Chinese Dongxiang group and the 30 comparison populations with the color scale ranging from light green to orange.

Nei’ *D_A_* distance is one of the most commonly used genetic distances to measure the genetic divergence between species or between populations within the same species and it was developed under the assumption that genetic differences originated from genetic drift and mutation events (Nei and Roychoudhury, 1974). Presently, the Nei’s *D_A_* distance was calculated and a heatmap of *D_A_* distance values was subsequently conducted to intuitively reflect the genetic relationships between Dongxiang and the 30 reference populations. As shown in **Figure 2**, the horizontal and vertical axis of the triangle were labeled by corresponding group names. And the color bar next to the triangle displayed the magnitude of *D_A_* values from 0.01 to 0.07, with the corresponding color ranging from light green to orange. The color of each block included in the triangle represented the *D_A_* values of pairwise populations and the one covered by darker color indicated a relative far genetic relation, and vice versa. Lighter color blocks were observed between Dongxiang and most Chinese groups, especially Tibet, Xibe, Tujia groups, and several Han populations, whereas darker color blocks were detected between Dongxiang and non-Chinese populations. Hence, observations indicated that Dongxiang group could be genetically closer related with Tibetan group in Qinghai (*D_A_* = 0.0021), several Han populations in Henan, Shanghai, Beijing, and Chengdu (*D_A_* = 0.0022, 0.0022, 0.0023, 0.0024), Xibe group (*D_A_* = 0.0024) and Tibetan group in Tibet (*D_A_* = 0.0028), and far related with the above-mentioned non-Chinese populations with the least *D_A_* distance values found between Dongxiang and Cape Colored population (*D_A_* = 0.0159).

**FIGURE 3:**
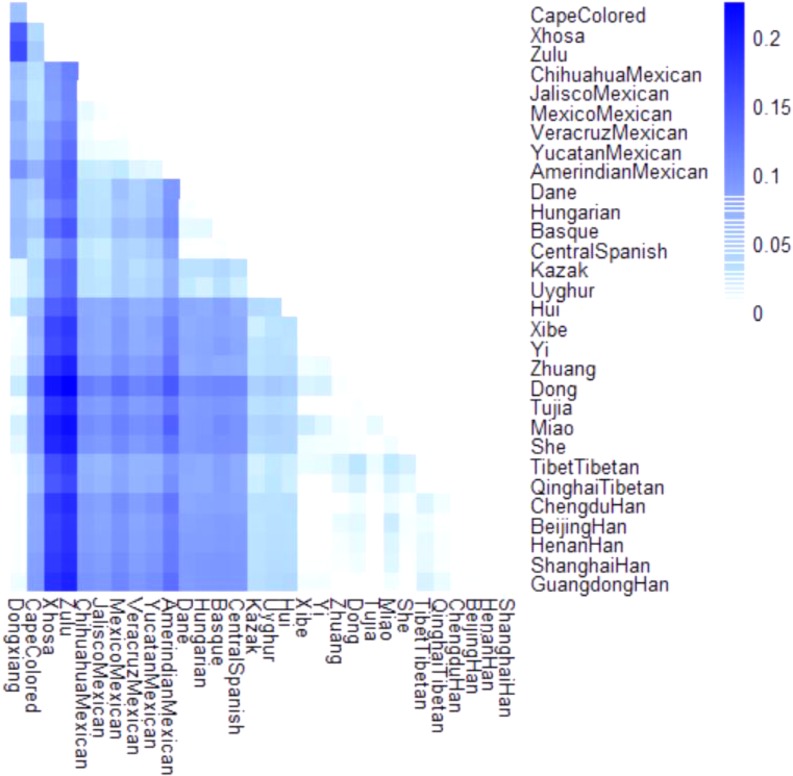
Heatmap of pairwise *F_st_* values for Chinese Dongxiang group and the 30 reference populations with the color scale ranging from white to dark blue.

The *F_st_* is generally considered as a measure of population differentiation on account of genetic structure (Jakobsson et al., 2013). A heatmap of *F_st_* values was also constructed in our study to mirror the differentiation degrees of pairwise populations. As demonstrated in **Figure 3**, the darker the block color was, the more the significant genetic differentiations existed between populations, and vice versa. And the color scale ranged from white to dark blue. It was visible that a set of blocks with lighter color exhibited between Dongxiang and most Chinese reference populations, which meant small genetic discrepancies existed between Dongxiang and these populations.

**FIGURE 4:**
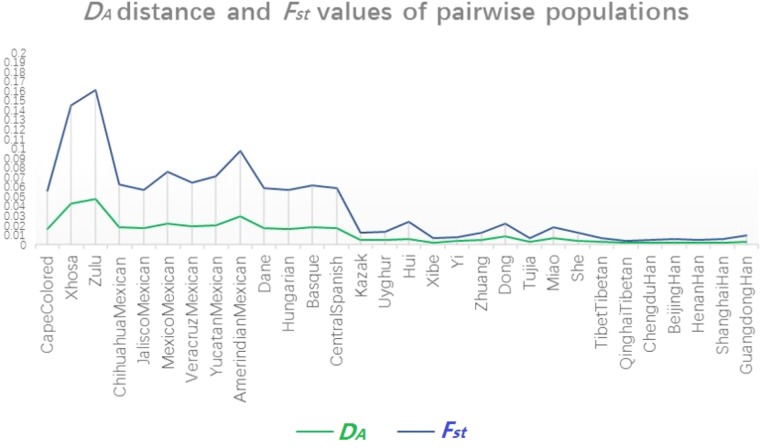
Multiple line chart of pairwise *D_A_* distance values and *F_st_* values for Chinese Dongxiang group and the 30 comparison populations.

To further illustrate the genetic relationships between Dongxiang and the other 30 populations, a multiplex line chart showing the variation tendency of *D_A_* distance and *F_st_* values was conducted by EXCEL spreadsheet 2016. As shown in **Figure 4**, the green line representing the change of *D_A_* distance values and the light blue line exhibiting the variation of *F_st_* values showed coincident change tendencies, indicating that the results were credible from another aspect. Detailed information about *D_A_* distance values and *F_st_* values was attached in **Supplementary Tables S3, S4**, respectively.

### Multidimensional Scaling Analyses Among the 31 Populations

**FIGURE 5:**
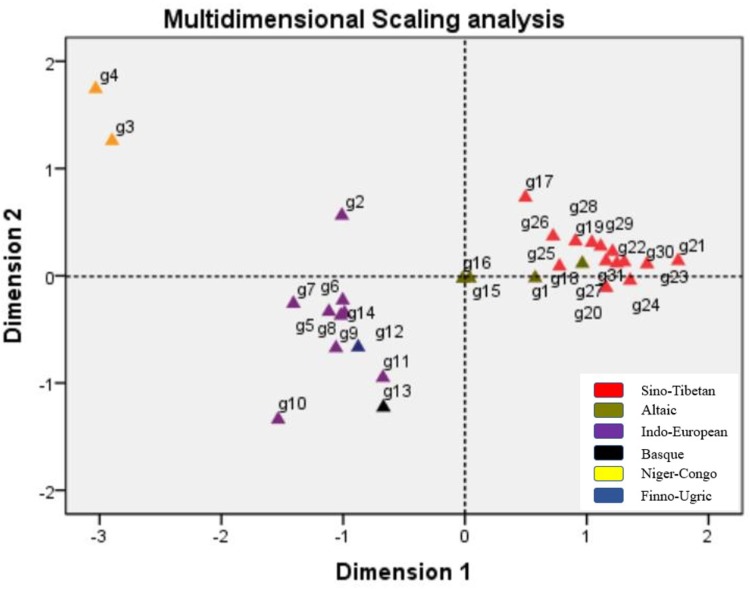
Multidimensional scaling analysis performed basing on pairwise *F_st_* values for Chinese Dongxiang group and the 30 reference populations (g1, Dongxiang; g2, Cape Colored; g3, Xhosa; g4, Zulu; g5, Chihuahua Mexican; g6, Jalisco Mexican; g7, Mexico Mexican; g8, Veracruz Mexican; g9, Yucatan Mexican; g10, Amerindian Mexican; g11, Dane; g12, Hungarian; g13, Basque; g14, Central Spanish; g15, Kazak; g16, Uyghur; g17, Hui; g18, Xibe; g19, Yi; g20, Zhuang; g21, Dong; g22, Tujia; g23, Miao; g24, She; g25, Tibet Tibetan; g26, Qinghai Tibetan; g27, Chengdu Han; g28, Beijing Han; g29, Henan Han; g30, Shanghai Han; g31, Guangdong Han).

Multidimensional scaling analysis is a generally employed method with the capability to visualize the similarity level of individual cases of a dataset. An MDS algorithm aims at placing each object in a N-dimensional space and the distances between two different objects can be preserved as well as possible (Borg and Groenen, 1997). Presently in our study, the MDS plot was constructed based on pairwise *F_st_* values to reflect the genetic relationships among 31 populations. As shown in **Figure 5**, all the 31 populations were exhibited with small icons and the colors were labeled according to their language families. It was noticeable that the population distributions in the plot were in general concordance with their geographic regions: all the East Asian populations involved in our study located at the right part of the plot, the only two Central Asian groups (Kazak and Uyghur) positioned in the middle of the plot and the left part of the plot was occupied by six Mexican groups, four European groups, and three African groups. Apparently, the studied Dongxiang group closely assembled with most Chinese populations (Tibetan in Tibet and Qinghai, Han populations in several different regions, Xibe, Hui, Tujia, Miao, Dong, and Zhuang), relatively far distant from two Central Asian populations (Uyghur, Kazak), six Mexican populations, and four European populations, most far distant from three African populations. So, MDS plot also verified that Dongxiang group was in close genetic relation to most Chinese populations, especially Tibet groups, Xibe group, and several Han populations.

### Population Substructure Analysis for Dongxiang and 30 Reference Populations

**FIGURE 6:**
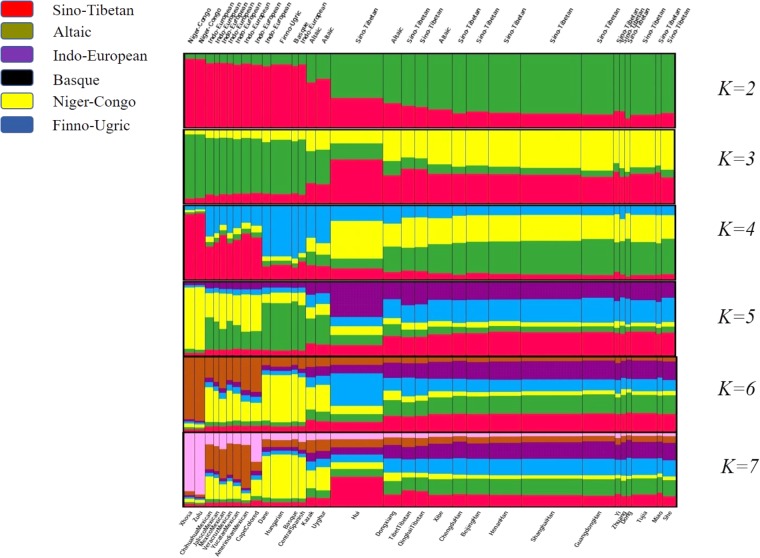
Structure clustering analysis conducted at population level based on genotyping data of the 30-InDel loci for the 31 populations by STRUCTURE version 2.3.4 software.

STRUCTURE analysis is commonly recognized to be capable of inferring population structure and assigning individuals to populations using multi-locus genotypic data (Pritchard et al., 2000). In present study, STRUCTURE clustering analysis was performed to reflect the memberships of biogeographic ancestry components for Dongxiang group and the reference populations with the number of hypothetic populations (*K*) defined at 2–7. And a burn-in period of 10,000 was also taken into account to acquire representative estimations of the parameters. As shown in **Figure 6**, population names as well as their corresponding language families were labeled on the bottom and the top of the figure. The width of each bar was proportional with the population sample size. When *K* at 2 and 3, East Asian groups and non-East Asian groups could be differentiated by distinct discrepancy of color compositions. And when *K* at 4, two African indigenous populations (Xhosa and Zulu), six Mexican groups (Chihuahua Mexican, Jalisco Mexican, Mexico Mexican, Veracruz Mexican, Yucatan Mexican, and Amerindian Mexican) and Cape Colored population, a subset of European groups (Dane, Hungarian, Basque, Central Spanish), and two Central Asian groups (Uyghur and Kazak) could be further distinguished. Similar clustering results could be generated at *K* = 5, 6. And when *K* = 7, Cape Colored population differed from Mexican groups with less brown components and more pink components. We surprisingly discovered the population substructure traits of Dongxiang group exerted similar hypothetical ancestry components with additional East Asian populations involved in our study at *K* = 3, 4, 5, 6, 7, which meant Dongxiang group was genetically closer with the most of Chinese populations involved in our study rather than other non-Chinese populations.

### Phylogenetic Reconstruction Generated Based on *D_A_* Distance Values and Allele Frequencies

**FIGURE 7:**
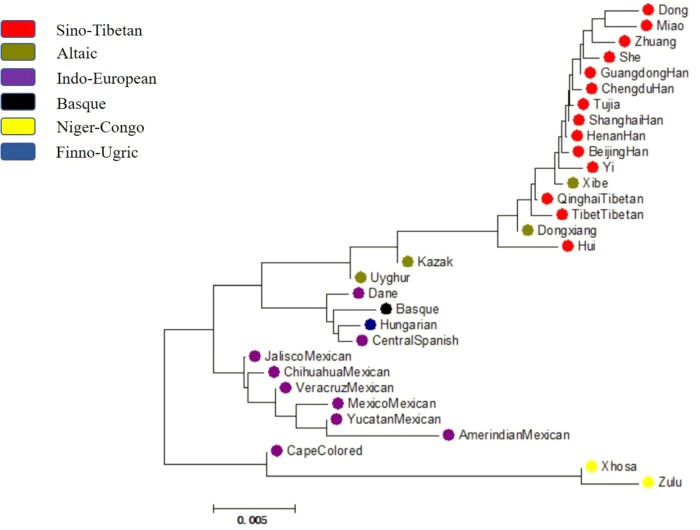
A phylogenetic tree conducted based upon *D_A_* distance values of Chinese Dongxiang group and the 30 comparison groups by MEGA version 6.06 software.

With neighbor-joining method applied, a phylogenetic tree was conducted based on *D_A_* values among Dongxiang and the 30 reference populations and displayed in **Figure 7**. The color of each population was labeled according to their corresponding language families. And four distinct branches were easily distinguished, with the first, second, third, and fourth branch composed of eighteen Asian populations, four European populations, six American populations, and three African populations, respectively. And we found clustering of the 31 populations roughly complied with their geographic locations and language families. The studied Dongxiang group was found to cluster with Tibet groups in Tibet and Qinghai, Xibe group, Hui group, and Han populations of diverse regions (Chengdu, Beijing, and Henan), indicating that relative close genetic relationships could be detectable among these populations. In exception of Cape Colored group, the only two African indigenous groups were discovered to be far related with most of the populations, which was in good accordance with previous studies. Furthermore, an unrooted tree (**Supplementary Figure S2**) was also constructed based on allele frequencies of the 30 loci by Phylip version 3.69 software, and the population distribution was quite similar with the above-mentioned mega tree, so we further validated our results.

Recently, the development of DNA genotyping technology provides a promising approach to explore the genetic backgrounds for Dongxiang group and fascinated the progress of origin exploration for Dongxiang group to a certain extent. Xie et al. (2002) conducted a phylogenetic tree for Dongxiang and its reference groups and reported the genetic similarities among Dongxiang, Hui, Tibetan, and Beijing Han populations. Moreover, Yao et al. (2016) reported that Dongxiang ethnic group displayed remarkable genetic homogeneity with Hans in Linxia and several additional East Asian populations. Obviously, researches cited above indicated the Dongxiang group might be closely related with Tibet group and Han populations, which was in agreement with our finding to a large extent. As we know, except for genetic markers, explorations of population-specific origins could be implemented from multiple aspects, such as languages, cultures, and so on. Dongxiang group is one of the Muslin groups of China. The language of Dongxiang ethnic group is a member of Mongolic family. Today, villagers residing in northeastern Dongxiang county also speak the “Tang Wang” language, which is a kind of creolized language recognized to be mixed by Mandarin and their original language. And surnames of Dongxiang people are also largely influenced by miscegenation phenomenon with the prevalence of Mongol, Han Chinese, and Tibetan surnames, like Wang, Kang, Zhang et al. (Howard, 1998). Similarly, humanity evidences identically indicated that relative frequent gene flow could be existed between Dongxiang and the adjacent Tibet group, Han population, which supported the close genetic relationships among these groups.

## Conclusion

At present, the forensic efficiency of the 30-InDel panel was assessed in Chinese Dongxiang ethnic group with the enrollment of 169 unrelated healthy individuals. And the results of CMP (3.96 × 10^-11^) and CPE (0.9886) certified the usefulness of these 30-InDel loci for forensic personal identification. Besides, to further clarify the genetic origin of Dongxiang ethnic group, we firstly applied the 30 insertion-deletion polymorphic genetic markers to explore the genetic relationships between the studied Dongxiang group and additional 30 reference populations. And observations indicated that Dongxiang was close related with Xibe group, Tibet groups in Tibet and Qinghai, and Han populations of several different regions (Chengdu, Beijing, and Henan). We believe our data presented here can be meaningful for further enriching the genetic background researches for Dongxiang group.

## Ethics Statement

This study was carried out according to the recommendations of “Human and Ethical Committee of Southern Medical University and Xi’an Jiaotong University, China” with written informed consent from all subjects. All subjects gave written informed consent in accordance with the Declaration of Helsinki. The protocol was approved by the “Human and Ethical Committee of Southern Medical University”.

## Author Contributions

BZ and XL designed this study. QL wrote the manuscript. YG, TX, YF, XJ, and WC collected the samples and extracted DNA. CC and YZ helped to conduct the statistical analysis. BZ also revised the manuscript. All authors agreed to the submission of the manuscript.

## Conflict of Interest Statement

The authors declare that the research was conducted in the absence of any commercial or financial relationships that could be construed as a potential conflict of interest.

## Footnotes

1 http://www.personal.psu.edu/nxm2/software.htm

2 http://www.megasoftware.net/

3 http://evolution.gs.washington.edu/phylip.html

## Supplementary Material

FIGURE S1 Pairwise LD analysis of for the 30 InDel loci in Chinese Dongxiang group by SNPAnalyzer version 2.0 software.

FIGURE S2 An unrooted phylogenetic tree constructed on the basis of allele frequencies of the 30 InDel loci by Phylip version 3.69 software.

TABLE S1 Several indices for LD including |D’|, r^2^ and LOD of pairwise InDel loci.

TABLE S2 *P* values of pairwise population differentiations between Dongxiang and the 30 reference populations.

TABLE S3 D_A_ distance values for Chinese Dongxiang group and the 30 reference populations.

TABLE S4 Pairwise F_st_ values for Chinese Dongxiang group and the reference populations.
