# Supplementary figures and images for: Population Genetic Diversity and Clustering Analysis for Chinese Dongxiang Group With 30 Autosomal InDel Loci Simultaneously Analyzed
Source: Front Genet. 2018 Aug 2;9:279. doi: 10.3389/fgene.2018.00279 (PMC6082941; doi:10.3389/fgene.2018.00279)

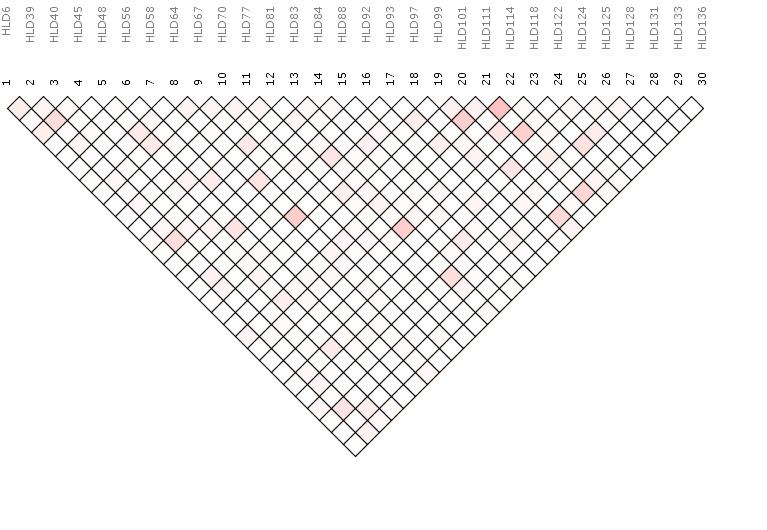

Supplement: FIGURE S1 — Pairwise LD analysis of for the 30 InDel loci in Chinese Dongxiang group by SNPAnalyzer version 2.0 software. [file Image_1.TIF]

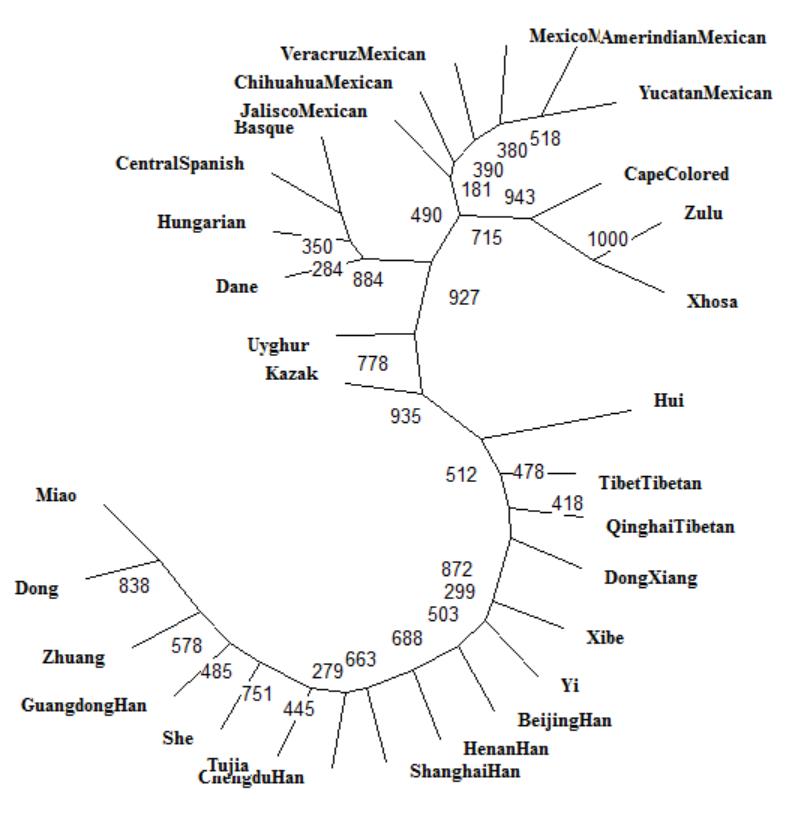

Supplement: FIGURE S2 — An unrooted phylogenetic tree constructed on the basis of allele frequencies of the 30 InDel loci by Phylip version 3.69 software. [file Image_2.JPEG]
