# Supplementary material for: Population Genetic Diversity and Clustering Analysis for Chinese Dongxiang Group With 30 Autosomal InDel Loci Simultaneously Analyzed
Source: Front Genet. 2018 Aug 2;9:279. doi: 10.3389/fgene.2018.00279 (PMC6082941; doi:10.3389/fgene.2018.00279)
Supplement: TABLE S1 — Several indices for LD including |D’|, r2 and LOD of pairwise InDel loci. [file Table_1.DOCX]

| Supplemental Table 1. Several indices for LD including \|D'\|, r^2^ and LOD of pairwise InDel loci. | | | | | | | |  |  |  |
| --- | --- | --- | --- | --- | --- | --- | --- | --- | --- | --- |
| No | Marker1 | Marker2 | Distance | \|D'\| | r^2^ | LOD | LOD_ p_ value | Chi_ square | p_ value | Four_ gamete |
| 1 | 0 | 1 | 0 | 0.2625 | 0.0183 | 2.9248 | 0.0872 | 6.1909 | 0.0128 | Y |
| 2 | 0 | 2 | 0 | 0.1337 | 0.0103 | 1.2480 | 0.2639 | 3.4629 | 0.0628 | Y |
| 3 | 0 | 3 | 0 | 0.2531 | 0.0298 | 6.1017 | 0.0135 | 10.0805 | 0.0015 | Y |
| 4 | 0 | 4 | 0 | 0.0291 | 0.0005 | 0.1550 | 0.6939 | 0.1841 | 0.6678 | Y |
| 5 | 0 | 5 | 0 | 0.0009 | 0.0000 | 0.0015 | 0.9688 | 0.0002 | 0.9886 | Y |
| 6 | 0 | 6 | 0 | 0.0073 | 0.0000 | 0.0012 | 0.9730 | 0.0121 | 0.9126 | Y |
| 7 | 0 | 7 | 0 | 0.1365 | 0.0049 | 1.0752 | 0.2998 | 1.6442 | 0.1998 | Y |
| 8 | 0 | 8 | 0 | 0.0490 | 0.0019 | 0.4392 | 0.5075 | 0.6468 | 0.4213 | Y |
| 9 | 0 | 9 | 0 | 0.0055 | 0.0000 | -0.0146 | 1.0000 | 0.0075 | 0.9309 | Y |
| 10 | 0 | 10 | 0 | 0.0348 | 0.0011 | 0.1592 | 0.6899 | 0.3589 | 0.5491 | Y |
| 11 | 0 | 11 | 0 | 0.0741 | 0.0016 | 0.1916 | 0.6616 | 0.5557 | 0.4560 | Y |
| 12 | 0 | 12 | 0 | 0.1370 | 0.0116 | 2.0914 | 0.1481 | 3.9202 | 0.0477 | Y |
| 13 | 0 | 13 | 0 | 0.1045 | 0.0055 | 1.2169 | 0.2700 | 1.8455 | 0.1743 | Y |
| 14 | 0 | 14 | 0 | 0.0146 | 0.0002 | 0.0694 | 0.7922 | 0.0671 | 0.7957 | Y |
| 15 | 0 | 15 | 0 | 0.0406 | 0.0010 | 0.1148 | 0.7347 | 0.3337 | 0.5635 | Y |
| 16 | 0 | 16 | 0 | 0.0125 | 0.0001 | -0.0514 | 1.0000 | 0.0419 | 0.8379 | Y |
| 17 | 0 | 17 | 0 | 0.1050 | 0.0066 | 0.9364 | 0.3332 | 2.2362 | 0.1348 | Y |
| 18 | 0 | 18 | 0 | 0.0241 | 0.0001 | -0.0137 | 1.0000 | 0.0345 | 0.8528 | Y |
| 19 | 0 | 19 | 0 | 0.0922 | 0.0074 | 1.0137 | 0.3140 | 2.4943 | 0.1143 | Y |
| 20 | 0 | 20 | 0 | 0.2034 | 0.0076 | 1.6213 | 0.2029 | 2.5663 | 0.1092 | Y |
| 21 | 0 | 21 | 0 | 0.0063 | 0.0000 | -0.0421 | 1.0000 | 0.0062 | 0.9374 | Y |
| 22 | 0 | 22 | 0 | 0.0010 | 0.0000 | 0.0003 | 0.9869 | 0.0001 | 0.9939 | Y |
| 23 | 0 | 23 | 0 | 0.0437 | 0.0009 | 0.2131 | 0.6444 | 0.2999 | 0.5839 | Y |
| 24 | 0 | 24 | 0 | 0.0964 | 0.0066 | 1.0182 | 0.3130 | 2.2193 | 0.1363 | Y |
| 25 | 0 | 25 | 0 | 0.0102 | 0.0001 | 0.0508 | 0.8217 | 0.0301 | 0.8622 | Y |
| 26 | 0 | 26 | 0 | 0.1753 | 0.0189 | 4.1167 | 0.0425 | 6.3932 | 0.0115 | Y |
| 27 | 0 | 27 | 0 | 0.1122 | 0.0092 | 1.6067 | 0.2050 | 3.1198 | 0.0773 | Y |
| 28 | 0 | 28 | 0 | 0.0775 | 0.0030 | 0.2894 | 0.5906 | 1.0243 | 0.3115 | Y |
| 29 | 0 | 29 | 0 | 0.0230 | 0.0005 | 0.0178 | 0.8940 | 0.1624 | 0.6870 | Y |
| 30 | 1 | 2 | 0 | 0.1993 | 0.0171 | 3.3305 | 0.0680 | 5.7917 | 0.0161 | Y |
| 31 | 1 | 3 | 0 | 0.3680 | 0.0168 | 3.6250 | 0.0569 | 5.6636 | 0.0173 | Y |
| 32 | 1 | 4 | 0 | 0.0932 | 0.0034 | 0.7578 | 0.3840 | 1.1332 | 0.2871 | Y |
| 33 | 1 | 5 | 0 | 0.1880 | 0.0066 | 1.0394 | 0.3080 | 2.2194 | 0.1363 | Y |
| 34 | 1 | 6 | 0 | 0.0722 | 0.0021 | 0.3732 | 0.5413 | 0.7061 | 0.4007 | Y |
| 35 | 1 | 7 | 0 | 0.0028 | 0.0000 | 0.0030 | 0.9567 | 0.0027 | 0.9588 | Y |
| 36 | 1 | 8 | 0 | 0.1694 | 0.0096 | 1.2565 | 0.2623 | 3.2313 | 0.0723 | Y |
| 37 | 1 | 9 | 0 | 0.0890 | 0.0027 | 0.4211 | 0.5164 | 0.9036 | 0.3418 | Y |
| 38 | 1 | 10 | 0 | 0.1661 | 0.0084 | 1.7630 | 0.1843 | 2.8228 | 0.0929 | Y |
| 39 | 1 | 11 | 0 | 0.0753 | 0.0005 | 0.1217 | 0.7272 | 0.1525 | 0.6962 | Y |
| 40 | 1 | 12 | 0 | 0.1683 | 0.0113 | 2.3224 | 0.1275 | 3.8333 | 0.0502 | Y |
| 41 | 1 | 13 | 0 | 0.3680 | 0.0168 | 3.6250 | 0.0569 | 5.6636 | 0.0173 | Y |
| 42 | 1 | 14 | 0 | 0.1167 | 0.0039 | 0.8495 | 0.3567 | 1.3142 | 0.2516 | Y |
| 43 | 1 | 15 | 0 | 0.1071 | 0.0051 | 1.0912 | 0.2962 | 1.7162 | 0.1902 | Y |
| 44 | 1 | 16 | 0 | 0.2275 | 0.0110 | 1.7496 | 0.1859 | 3.7093 | 0.0541 | Y |
| 45 | 1 | 17 | 0 | 0.2060 | 0.0063 | 1.3580 | 0.2439 | 2.1312 | 0.1443 | Y |
| 46 | 1 | 18 | 0 | 0.0009 | 0.0000 | 0.0003 | 0.9861 | 0.0000 | 0.9971 | Y |
| 47 | 1 | 19 | 0 | 0.0272 | 0.0002 | 0.0600 | 0.8065 | 0.0576 | 0.8104 | Y |
| 48 | 1 | 20 | 0 | 0.0767 | 0.0044 | 0.7000 | 0.4028 | 1.4752 | 0.2245 | Y |
| 49 | 1 | 21 | 0 | 0.1268 | 0.0018 | 0.3591 | 0.5490 | 0.6196 | 0.4312 | Y |
| 50 | 1 | 22 | 0 | 0.0617 | 0.0025 | 0.5116 | 0.4745 | 0.8511 | 0.3562 | Y |
| 51 | 1 | 23 | 0 | 0.0121 | 0.0001 | 0.0397 | 0.8421 | 0.0265 | 0.8708 | Y |
| 52 | 1 | 24 | 0 | 0.1657 | 0.0048 | 1.0867 | 0.2972 | 1.6225 | 0.2028 | Y |
| 53 | 1 | 25 | 0 | 0.1997 | 0.0124 | 1.4254 | 0.2325 | 4.1822 | 0.0409 | Y |
| 54 | 1 | 26 | 0 | 0.1220 | 0.0023 | 0.5368 | 0.4638 | 0.7670 | 0.3812 | Y |
| 55 | 1 | 27 | 0 | 0.3259 | 0.0207 | 4.3397 | 0.0372 | 7.0027 | 0.0081 | Y |
| 56 | 1 | 28 | 0 | 0.1026 | 0.0052 | 1.3362 | 0.2477 | 1.7450 | 0.1865 | Y |
| 57 | 1 | 29 | 0 | 0.2334 | 0.0132 | 2.6209 | 0.1055 | 4.4513 | 0.0349 | Y |
| 58 | 2 | 3 | 0 | 0.1358 | 0.0053 | 0.9302 | 0.3348 | 1.7860 | 0.1814 | Y |
| 59 | 2 | 4 | 0 | 0.0360 | 0.0005 | 0.1720 | 0.6783 | 0.1730 | 0.6774 | Y |
| 60 | 2 | 5 | 0 | 0.1288 | 0.0071 | 1.5132 | 0.2187 | 2.4141 | 0.1203 | Y |
| 61 | 2 | 6 | 0 | 0.0837 | 0.0065 | 0.8651 | 0.3523 | 2.1964 | 0.1383 | Y |
| 62 | 2 | 7 | 0 | 0.0094 | 0.0000 | 0.0344 | 0.8529 | 0.0127 | 0.9103 | Y |
| 63 | 2 | 8 | 0 | 0.1086 | 0.0054 | 1.4049 | 0.2359 | 1.8237 | 0.1769 | Y |
| 64 | 2 | 9 | 0 | 0.0065 | 0.0000 | -0.0257 | 1.0000 | 0.0111 | 0.9163 | Y |
| 65 | 2 | 10 | 0 | 0.0686 | 0.0024 | 0.2867 | 0.5923 | 0.7996 | 0.3712 | Y |
| 66 | 2 | 11 | 0 | 0.0969 | 0.0049 | 1.3428 | 0.2466 | 1.6554 | 0.1982 | Y |
| 67 | 2 | 12 | 0 | 0.0228 | 0.0002 | -0.0119 | 1.0000 | 0.0667 | 0.7962 | Y |
| 68 | 2 | 13 | 0 | 0.1670 | 0.0080 | 1.7427 | 0.1868 | 2.7027 | 0.1002 | Y |
| 69 | 2 | 14 | 0 | 0.0313 | 0.0007 | 0.2803 | 0.5965 | 0.2190 | 0.6398 | Y |
| 70 | 2 | 15 | 0 | 0.1019 | 0.0101 | 1.4815 | 0.2235 | 3.4197 | 0.0644 | Y |
| 71 | 2 | 16 | 0 | 0.0052 | 0.0000 | 0.0295 | 0.8637 | 0.0045 | 0.9465 | Y |
| 72 | 2 | 17 | 0 | 0.0132 | 0.0002 | -0.0530 | 1.0000 | 0.0578 | 0.8101 | Y |
| 73 | 2 | 18 | 0 | 0.0254 | 0.0002 | -0.0142 | 1.0000 | 0.0669 | 0.7959 | Y |
| 74 | 2 | 19 | 0 | 0.0291 | 0.0006 | 0.0672 | 0.7955 | 0.1889 | 0.6639 | Y |
| 75 | 2 | 20 | 0 | 0.0834 | 0.0022 | 0.1289 | 0.7195 | 0.7528 | 0.3856 | Y |
| 76 | 2 | 21 | 0 | 0.1436 | 0.0154 | 1.8596 | 0.1727 | 5.2150 | 0.0224 | Y |
| 77 | 2 | 22 | 0 | 0.1092 | 0.0012 | 0.2814 | 0.5958 | 0.4064 | 0.5238 | Y |
| 78 | 2 | 23 | 0 | 0.1262 | 0.0129 | 1.8397 | 0.1750 | 4.3727 | 0.0365 | Y |
| 79 | 2 | 24 | 0 | 0.0495 | 0.0010 | 0.2541 | 0.6142 | 0.3349 | 0.5628 | Y |
| 80 | 2 | 25 | 0 | 0.0704 | 0.0036 | 0.3031 | 0.5819 | 1.2022 | 0.2729 | Y |
| 81 | 2 | 26 | 0 | 0.0080 | 0.0000 | 0.0151 | 0.9021 | 0.0077 | 0.9303 | Y |
| 82 | 2 | 27 | 0 | 0.0738 | 0.0025 | 0.5831 | 0.4451 | 0.8320 | 0.3617 | Y |
| 83 | 2 | 28 | 0 | 0.2436 | 0.0522 | 10.021 | 0.0016 | 17.640 | 0.0000 | Y |
| 84 | 2 | 29 | 0 | 0.1698 | 0.0161 | 1.5980 | 0.2062 | 5.4567 | 0.0195 | Y |
| 85 | 3 | 4 | 0 | 0.0205 | 0.0001 | 0.0748 | 0.7845 | 0.0456 | 0.8310 | Y |
| 86 | 3 | 5 | 0 | 0.0152 | 0.0002 | 0.0567 | 0.8118 | 0.0517 | 0.8202 | Y |
| 87 | 3 | 6 | 0 | 0.0823 | 0.0051 | 0.4671 | 0.4943 | 1.7245 | 0.1891 | Y |
| 88 | 3 | 7 | 0 | 0.0981 | 0.0012 | -0.1164 | 1.0000 | 0.3948 | 0.5298 | Y |
| 89 | 3 | 8 | 0 | 0.0399 | 0.0010 | 0.2658 | 0.6061 | 0.3374 | 0.5613 | Y |
| 90 | 3 | 9 | 0 | 0.0217 | 0.0003 | 0.0211 | 0.8844 | 0.1010 | 0.7506 | Y |
| 91 | 3 | 10 | 0 | 0.0831 | 0.0039 | 0.6535 | 0.4189 | 1.3288 | 0.2490 | Y |
| 92 | 3 | 11 | 0 | 0.1524 | 0.0149 | 2.9498 | 0.0859 | 5.0436 | 0.0247 | Y |
| 93 | 3 | 12 | 0 | 0.0474 | 0.0017 | 0.2477 | 0.6187 | 0.5720 | 0.4495 | Y |
| 94 | 3 | 13 | 0 | 0.0872 | 0.0076 | 1.6226 | 0.2027 | 2.5671 | 0.1091 | Y |
| 95 | 3 | 14 | 0 | 0.1992 | 0.0172 | 3.0354 | 0.0815 | 5.8153 | 0.0159 | Y |
| 96 | 3 | 15 | 0 | 0.0934 | 0.0073 | 1.3362 | 0.2477 | 2.4544 | 0.1172 | Y |
| 97 | 3 | 16 | 0 | 0.1297 | 0.0067 | 0.9331 | 0.3341 | 2.2702 | 0.1319 | Y |
| 98 | 3 | 17 | 0 | 0.1255 | 0.0044 | 1.2385 | 0.2658 | 1.4874 | 0.2226 | Y |
| 99 | 3 | 18 | 0 | 0.1780 | 0.0028 | 0.5388 | 0.4630 | 0.9398 | 0.3323 | Y |
| 100 | 3 | 19 | 0 | 0.1390 | 0.0104 | 1.7016 | 0.1921 | 3.5034 | 0.0612 | Y |
| 101 | 3 | 20 | 0 | 0.2630 | 0.0273 | 4.1726 | 0.0411 | 9.2152 | 0.0024 | Y |
| 102 | 3 | 21 | 0 | 0.0086 | 0.0001 | -0.0374 | 1.0000 | 0.0231 | 0.8793 | Y |
| 103 | 3 | 22 | 0 | 0.0343 | 0.0004 | 0.0600 | 0.8065 | 0.1401 | 0.7082 | Y |
| 104 | 3 | 23 | 0 | 0.0843 | 0.0017 | 0.6014 | 0.4381 | 0.5589 | 0.4547 | Y |
| 105 | 3 | 24 | 0 | 0.2859 | 0.0269 | 5.5507 | 0.0185 | 9.0836 | 0.0026 | Y |
| 106 | 3 | 25 | 0 | 0.0737 | 0.0022 | 0.2129 | 0.6445 | 0.7325 | 0.3921 | Y |
| 107 | 3 | 26 | 0 | 0.0097 | 0.0001 | -0.0149 | 1.0000 | 0.0256 | 0.8730 | Y |
| 108 | 3 | 27 | 0 | 0.0119 | 0.0001 | 0.0216 | 0.8831 | 0.0174 | 0.8949 | Y |
| 109 | 3 | 28 | 0 | 0.0036 | 0.0000 | -0.0094 | 1.0000 | 0.0040 | 0.9496 | Y |
| 110 | 3 | 29 | 0 | 0.1237 | 0.0070 | 0.9317 | 0.3344 | 2.3514 | 0.1252 | Y |
| 111 | 4 | 5 | 0 | 0.1244 | 0.0075 | 1.0261 | 0.3111 | 2.5200 | 0.1124 | Y |
| 112 | 4 | 6 | 0 | 0.0224 | 0.0002 | -0.0085 | 1.0000 | 0.0719 | 0.7886 | Y |
| 113 | 4 | 7 | 0 | 0.2990 | 0.0150 | 3.7774 | 0.0520 | 5.0621 | 0.0245 | Y |
| 114 | 4 | 8 | 0 | 0.2994 | 0.0459 | 8.3714 | 0.0038 | 15.507 | 0.0001 | Y |
| 115 | 4 | 9 | 0 | 0.0608 | 0.0032 | 0.7400 | 0.3897 | 1.0942 | 0.2955 | Y |
| 116 | 4 | 10 | 0 | 0.0496 | 0.0019 | 0.3431 | 0.5580 | 0.6517 | 0.4195 | Y |
| 117 | 4 | 11 | 0 | 0.2143 | 0.0095 | 2.1699 | 0.1407 | 3.1997 | 0.0737 | Y |
| 118 | 4 | 12 | 0 | 0.0574 | 0.0032 | 0.4705 | 0.4928 | 1.0718 | 0.3005 | Y |
| 119 | 4 | 13 | 0 | 0.0830 | 0.0050 | 1.0851 | 0.2976 | 1.6874 | 0.1939 | Y |
| 120 | 4 | 14 | 0 | 0.0197 | 0.0002 | 0.0654 | 0.7982 | 0.0781 | 0.7799 | Y |
| 121 | 4 | 15 | 0 | 0.3297 | 0.0419 | 7.5572 | 0.0060 | 14.1568 | 0.0002 | Y |
| 122 | 4 | 16 | 0 | 0.0551 | 0.0024 | 0.6044 | 0.4369 | 0.8258 | 0.3635 | Y |
| 123 | 4 | 17 | 0 | 0.1606 | 0.0099 | 1.0377 | 0.3084 | 3.3584 | 0.0669 | Y |
| 124 | 4 | 18 | 0 | 0.0175 | 0.0001 | 0.0624 | 0.8027 | 0.0282 | 0.8668 | Y |
| 125 | 4 | 19 | 0 | 0.0328 | 0.0006 | 0.1844 | 0.6677 | 0.2168 | 0.6415 | Y |
| 126 | 4 | 20 | 0 | 0.1388 | 0.0055 | 1.3378 | 0.2474 | 1.8610 | 0.1725 | Y |
| 127 | 4 | 21 | 0 | 0.1599 | 0.0171 | 4.0987 | 0.0429 | 5.7749 | 0.0163 | Y |
| 128 | 4 | 22 | 0 | 0.0067 | 0.0000 | 0.0008 | 0.9777 | 0.0017 | 0.9671 | Y |
| 129 | 4 | 23 | 0 | 0.0301 | 0.0007 | 0.2172 | 0.6412 | 0.2222 | 0.6374 | Y |
| 130 | 4 | 24 | 0 | 0.1171 | 0.0062 | 1.1196 | 0.2900 | 2.1012 | 0.1472 | Y |
| 131 | 4 | 25 | 0 | 0.1085 | 0.0065 | 1.0101 | 0.3149 | 2.1894 | 0.1390 | Y |
| 132 | 4 | 26 | 0 | 0.0841 | 0.0063 | 1.6364 | 0.2008 | 2.1383 | 0.1437 | Y |
| 133 | 4 | 27 | 0 | 0.1082 | 0.0102 | 2.2842 | 0.1307 | 3.4625 | 0.0628 | Y |
| 134 | 4 | 28 | 0 | 0.0163 | 0.0002 | -0.0082 | 1.0000 | 0.0708 | 0.7901 | Y |
| 135 | 4 | 29 | 0 | 0.0708 | 0.0031 | 0.6351 | 0.4255 | 1.0613 | 0.3029 | Y |
| 136 | 5 | 6 | 0 | 0.0780 | 0.0028 | 0.4096 | 0.5222 | 0.9558 | 0.3283 | Y |
| 137 | 5 | 7 | 0 | 0.1772 | 0.0109 | 2.2782 | 0.1312 | 3.6905 | 0.0547 | Y |
| 138 | 5 | 8 | 0 | 0.0560 | 0.0030 | 0.2796 | 0.5970 | 0.9989 | 0.3176 | Y |
| 139 | 5 | 9 | 0 | 0.1295 | 0.0092 | 1.9179 | 0.1661 | 3.1228 | 0.0772 | Y |
| 140 | 5 | 10 | 0 | 0.0375 | 0.0012 | 0.2829 | 0.5948 | 0.4061 | 0.5240 | Y |
| 141 | 5 | 11 | 0 | 0.1055 | 0.0048 | 1.0945 | 0.2955 | 1.6089 | 0.2047 | Y |
| 142 | 5 | 12 | 0 | 0.2793 | 0.0362 | 6.8152 | 0.0090 | 12.2360 | 0.0005 | Y |
| 143 | 5 | 13 | 0 | 0.0220 | 0.0002 | -0.0047 | 1.0000 | 0.0571 | 0.8112 | Y |
| 144 | 5 | 14 | 0 | 0.0523 | 0.0022 | 0.4592 | 0.4980 | 0.7446 | 0.3882 | Y |
| 145 | 5 | 15 | 0 | 0.0149 | 0.0002 | 0.0633 | 0.8013 | 0.0602 | 0.8063 | Y |
| 146 | 5 | 16 | 0 | 0.0717 | 0.0031 | 0.6873 | 0.4071 | 1.0420 | 0.3074 | Y |
| 147 | 5 | 17 | 0 | 0.0323 | 0.0008 | 0.3273 | 0.5673 | 0.2822 | 0.5953 | Y |
| 148 | 5 | 18 | 0 | 0.1236 | 0.0020 | 0.5120 | 0.4743 | 0.6798 | 0.4096 | Y |
| 149 | 5 | 19 | 0 | 0.0314 | 0.0008 | 0.1323 | 0.7161 | 0.2692 | 0.6039 | Y |
| 150 | 5 | 20 | 0 | 0.1466 | 0.0056 | 1.1870 | 0.2759 | 1.9059 | 0.1674 | Y |
| 151 | 5 | 21 | 0 | 0.0573 | 0.0011 | 0.0238 | 0.8774 | 0.3578 | 0.5497 | Y |
| 152 | 5 | 22 | 0 | 0.0385 | 0.0002 | 0.0477 | 0.8271 | 0.0614 | 0.8042 | Y |
| 153 | 5 | 23 | 0 | 0.1128 | 0.0045 | 0.5265 | 0.4681 | 1.5032 | 0.2202 | Y |
| 154 | 5 | 24 | 0 | 0.0615 | 0.0019 | 0.4775 | 0.4896 | 0.6312 | 0.4269 | Y |
| 155 | 5 | 25 | 0 | 0.1287 | 0.0099 | 1.8085 | 0.1787 | 3.3534 | 0.0671 | Y |
| 156 | 5 | 26 | 0 | 0.0554 | 0.0013 | 0.3412 | 0.5592 | 0.4467 | 0.5039 | Y |
| 157 | 5 | 27 | 0 | 0.1043 | 0.0104 | 1.9258 | 0.1652 | 3.5037 | 0.0612 | Y |
| 158 | 5 | 28 | 0 | 0.0171 | 0.0001 | 0.0073 | 0.9321 | 0.0375 | 0.8464 | Y |
| 159 | 5 | 29 | 0 | 0.0731 | 0.0036 | 0.8163 | 0.3663 | 1.2317 | 0.2671 | Y |
| 160 | 6 | 7 | 0 | 0.0428 | 0.0003 | 0.1350 | 0.7133 | 0.0999 | 0.7520 | Y |
| 161 | 6 | 8 | 0 | 0.0125 | 0.0001 | 0.0307 | 0.8608 | 0.0442 | 0.8335 | Y |
| 162 | 6 | 9 | 0 | 0.0045 | 0.0000 | 0.0138 | 0.9065 | 0.0057 | 0.9398 | Y |
| 163 | 6 | 10 | 0 | 0.0716 | 0.0028 | 0.5835 | 0.4449 | 0.9413 | 0.3320 | Y |
| 164 | 6 | 11 | 0 | 0.0768 | 0.0012 | 0.3257 | 0.5682 | 0.3962 | 0.5291 | Y |
| 165 | 6 | 12 | 0 | 0.0772 | 0.0060 | 1.1062 | 0.2929 | 2.0153 | 0.1557 | Y |
| 166 | 6 | 13 | 0 | 0.1048 | 0.0083 | 1.7623 | 0.1843 | 2.7935 | 0.0947 | Y |
| 167 | 6 | 14 | 0 | 0.0073 | 0.0000 | -0.0061 | 1.0000 | 0.0127 | 0.9102 | Y |
| 168 | 6 | 15 | 0 | 0.0789 | 0.0056 | 1.2297 | 0.2675 | 1.9029 | 0.1678 | Y |
| 169 | 6 | 16 | 0 | 0.1179 | 0.0074 | 1.1543 | 0.2827 | 2.4909 | 0.1145 | Y |
| 170 | 6 | 17 | 0 | 0.0381 | 0.0005 | -0.0006 | 1.0000 | 0.1826 | 0.6692 | Y |
| 171 | 6 | 18 | 0 | 0.0613 | 0.0004 | 0.1196 | 0.7295 | 0.1479 | 0.7005 | Y |
| 172 | 6 | 19 | 0 | 0.0624 | 0.0022 | 0.5301 | 0.4666 | 0.7573 | 0.3842 | Y |
| 173 | 6 | 20 | 0 | 0.1119 | 0.0037 | 1.0283 | 0.3106 | 1.2564 | 0.2623 | Y |
| 174 | 6 | 21 | 0 | 0.1582 | 0.0071 | 1.1081 | 0.2925 | 2.4105 | 0.1205 | Y |
| 175 | 6 | 22 | 0 | 0.0262 | 0.0002 | 0.0217 | 0.8829 | 0.0615 | 0.8042 | Y |
| 176 | 6 | 23 | 0 | 0.0007 | 0.0000 | -0.0019 | 1.0000 | 0.0001 | 0.9941 | Y |
| 177 | 6 | 24 | 0 | 0.0790 | 0.0027 | 0.4173 | 0.5183 | 0.9215 | 0.3371 | Y |
| 178 | 6 | 25 | 0 | 0.1126 | 0.0098 | 2.1298 | 0.1445 | 3.3181 | 0.0685 | Y |
| 179 | 6 | 26 | 0 | 0.0614 | 0.0035 | 0.8079 | 0.3687 | 1.1810 | 0.2772 | Y |
| 180 | 6 | 27 | 0 | 0.1420 | 0.0098 | 2.1233 | 0.1451 | 3.3200 | 0.0684 | Y |
| 181 | 6 | 28 | 0 | 0.0632 | 0.0033 | 0.5672 | 0.4514 | 1.1013 | 0.2940 | Y |
| 182 | 6 | 29 | 0 | 0.1708 | 0.0198 | 2.9837 | 0.0841 | 6.7050 | 0.0096 | Y |
| 183 | 7 | 8 | 0 | 0.2256 | 0.0167 | 3.3135 | 0.0687 | 5.6270 | 0.0177 | Y |
| 184 | 7 | 9 | 0 | 0.0407 | 0.0006 | 0.0595 | 0.8074 | 0.1854 | 0.6668 | Y |
| 185 | 7 | 10 | 0 | 0.1232 | 0.0032 | 0.6423 | 0.4229 | 1.0943 | 0.2955 | Y |
| 186 | 7 | 11 | 0 | 0.0221 | 0.0004 | 0.2742 | 0.6005 | 0.1341 | 0.7143 | Y |
| 187 | 7 | 12 | 0 | 0.0341 | 0.0005 | 0.1196 | 0.7295 | 0.1546 | 0.6942 | Y |
| 188 | 7 | 13 | 0 | 0.0720 | 0.0027 | 0.5250 | 0.4687 | 0.9139 | 0.3391 | Y |
| 189 | 7 | 14 | 0 | 0.3159 | 0.0226 | 3.8882 | 0.0486 | 7.6381 | 0.0057 | Y |
| 190 | 7 | 15 | 0 | 0.1168 | 0.0059 | 0.4311 | 0.5114 | 2.0047 | 0.1568 | Y |
| 191 | 7 | 16 | 0 | 0.0009 | 0.0000 | -0.0015 | 1.0000 | 0.0001 | 0.9925 | Y |
| 192 | 7 | 17 | 0 | 0.4408 | 0.0284 | 5.9214 | 0.0150 | 9.5828 | 0.0020 | Y |
| 193 | 7 | 18 | 0 | 0.0031 | 0.0000 | 0.0003 | 0.9864 | 0.0024 | 0.9611 | Y |
| 194 | 7 | 19 | 0 | 0.0531 | 0.0006 | 0.0900 | 0.7642 | 0.2160 | 0.6421 | Y |
| 195 | 7 | 20 | 0 | 0.1905 | 0.0017 | 0.4824 | 0.4874 | 0.5876 | 0.4434 | Y |
| 196 | 7 | 21 | 0 | 0.1738 | 0.0171 | 2.0140 | 0.1559 | 5.7882 | 0.0161 | Y |
| 197 | 7 | 22 | 0 | 0.0269 | 0.0005 | 0.0998 | 0.7521 | 0.1647 | 0.6849 | Y |
| 198 | 7 | 23 | 0 | 0.1424 | 0.0025 | 0.6638 | 0.4152 | 0.8333 | 0.3613 | Y |
| 199 | 7 | 24 | 0 | 0.1413 | 0.0034 | 0.6813 | 0.4092 | 1.1586 | 0.2818 | Y |
| 200 | 7 | 25 | 0 | 0.1059 | 0.0034 | 0.6377 | 0.4245 | 1.1552 | 0.2825 | Y |
| 201 | 7 | 26 | 0 | 0.0686 | 0.0020 | 0.0201 | 0.8873 | 0.6731 | 0.4120 | Y |
| 202 | 7 | 27 | 0 | 0.0722 | 0.0017 | 0.3998 | 0.5272 | 0.5832 | 0.4451 | Y |
| 203 | 7 | 28 | 0 | 0.0518 | 0.0004 | 0.0159 | 0.8996 | 0.1191 | 0.7300 | Y |
| 204 | 7 | 29 | 0 | 0.0681 | 0.0012 | 0.5322 | 0.4657 | 0.4182 | 0.5178 | Y |
| 205 | 8 | 9 | 0 | 0.2047 | 0.0414 | 7.1483 | 0.0075 | 13.9960 | 0.0002 | Y |
| 206 | 8 | 10 | 0 | 0.0090 | 0.0001 | 0.0269 | 0.8698 | 0.0249 | 0.8745 | Y |
| 207 | 8 | 11 | 0 | 0.0153 | 0.0001 | -0.0001 | 1.0000 | 0.0319 | 0.8583 | Y |
| 208 | 8 | 12 | 0 | 0.2991 | 0.0441 | 7.9601 | 0.0048 | 14.9163 | 0.0001 | Y |
| 209 | 8 | 13 | 0 | 0.0942 | 0.0056 | 0.9247 | 0.3363 | 1.8817 | 0.1701 | Y |
| 210 | 8 | 14 | 0 | 0.0638 | 0.0035 | 0.7088 | 0.3998 | 1.1783 | 0.2777 | Y |
| 211 | 8 | 15 | 0 | 0.0096 | 0.0001 | 0.0269 | 0.8697 | 0.0233 | 0.8787 | Y |
| 212 | 8 | 16 | 0 | 0.0086 | 0.0001 | 0.0503 | 0.8226 | 0.0159 | 0.8997 | Y |
| 213 | 8 | 17 | 0 | 0.0991 | 0.0044 | 0.6163 | 0.4324 | 1.4810 | 0.2236 | Y |
| 214 | 8 | 18 | 0 | 0.0192 | 0.0001 | -0.0258 | 1.0000 | 0.0295 | 0.8637 | Y |
| 215 | 8 | 19 | 0 | 0.0722 | 0.0036 | 0.6539 | 0.4187 | 1.2202 | 0.2693 | Y |
| 216 | 8 | 20 | 0 | 0.1677 | 0.0070 | 1.2946 | 0.2552 | 2.3495 | 0.1253 | Y |
| 217 | 8 | 21 | 0 | 0.0938 | 0.0030 | 0.3181 | 0.5728 | 1.0164 | 0.3134 | Y |
| 218 | 8 | 22 | 0 | 0.0956 | 0.0012 | 0.2551 | 0.6135 | 0.4034 | 0.5253 | Y |
| 219 | 8 | 23 | 0 | 0.0943 | 0.0033 | 0.5192 | 0.4712 | 1.1160 | 0.2908 | Y |
| 220 | 8 | 24 | 0 | 0.0324 | 0.0006 | 0.1181 | 0.7311 | 0.1859 | 0.6664 | Y |
| 221 | 8 | 25 | 0 | 0.0052 | 0.0000 | -0.0010 | 1.0000 | 0.0059 | 0.9389 | Y |
| 222 | 8 | 26 | 0 | 0.1061 | 0.0052 | 0.9787 | 0.3225 | 1.7414 | 0.1870 | Y |
| 223 | 8 | 27 | 0 | 0.0052 | 0.0000 | 0.0165 | 0.8978 | 0.0089 | 0.9248 | Y |
| 224 | 8 | 28 | 0 | 0.1324 | 0.0070 | 1.1638 | 0.2807 | 2.3811 | 0.1228 | Y |
| 225 | 8 | 29 | 0 | 0.0088 | 0.0001 | -0.0315 | 1.0000 | 0.0214 | 0.8836 | Y |
| 226 | 9 | 10 | 0 | 0.2176 | 0.0305 | 6.0292 | 0.0141 | 10.3102 | 0.0013 | Y |
| 227 | 9 | 11 | 0 | 0.1116 | 0.0029 | 0.7625 | 0.3826 | 0.9927 | 0.3191 | Y |
| 228 | 9 | 12 | 0 | 0.0217 | 0.0004 | 0.1765 | 0.6744 | 0.1347 | 0.7137 | Y |
| 229 | 9 | 13 | 0 | 0.1331 | 0.0065 | 1.4058 | 0.2358 | 2.1971 | 0.1383 | Y |
| 230 | 9 | 14 | 0 | 0.0247 | 0.0005 | 0.0687 | 0.7932 | 0.1745 | 0.6761 | Y |
| 231 | 9 | 15 | 0 | 0.1093 | 0.0053 | 0.8952 | 0.3441 | 1.7776 | 0.1825 | Y |
| 232 | 9 | 16 | 0 | 0.0840 | 0.0044 | 1.0103 | 0.3148 | 1.5006 | 0.2206 | Y |
| 233 | 9 | 17 | 0 | 0.1017 | 0.0046 | 0.6315 | 0.4268 | 1.5400 | 0.2146 | Y |
| 234 | 9 | 18 | 0 | 0.0293 | 0.0002 | -0.0261 | 1.0000 | 0.0691 | 0.7926 | Y |
| 235 | 9 | 19 | 0 | 0.0222 | 0.0003 | 0.1091 | 0.7412 | 0.1139 | 0.7358 | Y |
| 236 | 9 | 20 | 0 | 0.1427 | 0.0029 | 0.3873 | 0.5337 | 0.9951 | 0.3185 | Y |
| 237 | 9 | 21 | 0 | 0.1674 | 0.0095 | 0.5268 | 0.4680 | 3.2027 | 0.0735 | Y |
| 238 | 9 | 22 | 0 | 0.0999 | 0.0022 | 0.4905 | 0.4837 | 0.7519 | 0.3859 | Y |
| 239 | 9 | 23 | 0 | 0.0438 | 0.0012 | 0.3705 | 0.5428 | 0.4117 | 0.5211 | Y |
| 240 | 9 | 24 | 0 | 0.0221 | 0.0004 | -0.0020 | 1.0000 | 0.1485 | 0.7000 | Y |
| 241 | 9 | 25 | 0 | 0.1204 | 0.0091 | 2.0380 | 0.1534 | 3.0789 | 0.0793 | Y |
| 242 | 9 | 26 | 0 | 0.0077 | 0.0001 | -0.0040 | 1.0000 | 0.0157 | 0.9004 | Y |
| 243 | 9 | 27 | 0 | 0.0649 | 0.0042 | 0.6132 | 0.4336 | 1.4218 | 0.2331 | Y |
| 244 | 9 | 28 | 0 | 0.0822 | 0.0027 | 0.3243 | 0.5690 | 0.9069 | 0.3409 | Y |
| 245 | 9 | 29 | 0 | 0.0135 | 0.0002 | -0.0559 | 1.0000 | 0.0495 | 0.8240 | Y |
| 246 | 10 | 11 | 0 | 0.1778 | 0.0083 | 1.7870 | 0.1813 | 2.8052 | 0.0940 | Y |
| 247 | 10 | 12 | 0 | 0.0177 | 0.0002 | 0.0785 | 0.7793 | 0.0575 | 0.8104 | Y |
| 248 | 10 | 13 | 0 | 0.0719 | 0.0029 | 0.6226 | 0.4301 | 0.9940 | 0.3188 | Y |
| 249 | 10 | 14 | 0 | 0.0418 | 0.0013 | 0.2768 | 0.5988 | 0.4494 | 0.5026 | Y |
| 250 | 10 | 15 | 0 | 0.1452 | 0.0144 | 2.4872 | 0.1148 | 4.8769 | 0.0272 | Y |
| 251 | 10 | 16 | 0 | 0.0670 | 0.0044 | 0.9575 | 0.3278 | 1.4806 | 0.2237 | Y |
| 252 | 10 | 17 | 0 | 0.0217 | 0.0002 | 0.0614 | 0.8043 | 0.0784 | 0.7795 | Y |
| 253 | 10 | 18 | 0 | 0.2545 | 0.0100 | 1.5242 | 0.2170 | 3.3705 | 0.0664 | Y |
| 254 | 10 | 19 | 0 | 0.1591 | 0.0239 | 5.1849 | 0.0228 | 8.0616 | 0.0045 | Y |
| 255 | 10 | 20 | 0 | 0.0128 | 0.0000 | 0.0093 | 0.9233 | 0.0089 | 0.9247 | Y |
| 256 | 10 | 21 | 0 | 0.1031 | 0.0040 | 1.0717 | 0.3006 | 1.3527 | 0.2448 | Y |
| 257 | 10 | 22 | 0 | 0.1243 | 0.0022 | 0.5476 | 0.4593 | 0.7505 | 0.3863 | Y |
| 258 | 10 | 23 | 0 | 0.0262 | 0.0004 | 0.0195 | 0.8890 | 0.1317 | 0.7167 | Y |
| 259 | 10 | 24 | 0 | 0.0822 | 0.0039 | 0.7447 | 0.3882 | 1.3169 | 0.2512 | Y |
| 260 | 10 | 25 | 0 | 0.0228 | 0.0005 | 0.0754 | 0.7837 | 0.1720 | 0.6784 | Y |
| 261 | 10 | 26 | 0 | 0.0322 | 0.0007 | 0.1271 | 0.7215 | 0.2451 | 0.6206 | Y |
| 262 | 10 | 27 | 0 | 0.0783 | 0.0040 | 0.9269 | 0.3357 | 1.3361 | 0.2477 | Y |
| 263 | 10 | 28 | 0 | 0.0326 | 0.0005 | 0.1403 | 0.7080 | 0.1594 | 0.6897 | Y |
| 264 | 10 | 29 | 0 | 0.0243 | 0.0005 | 0.1347 | 0.7137 | 0.1791 | 0.6722 | Y |
| 265 | 11 | 12 | 0 | 0.0511 | 0.0013 | 0.3481 | 0.5552 | 0.4272 | 0.5134 | Y |
| 266 | 11 | 13 | 0 | 0.2013 | 0.0061 | 1.5011 | 0.2205 | 2.0494 | 0.1523 | Y |
| 267 | 11 | 14 | 0 | 0.0312 | 0.0003 | 0.1299 | 0.7185 | 0.1133 | 0.7364 | Y |
| 268 | 11 | 15 | 0 | 0.1567 | 0.0044 | 0.9870 | 0.3205 | 1.4904 | 0.2222 | Y |
| 269 | 11 | 16 | 0 | 0.3158 | 0.0256 | 5.8080 | 0.0160 | 8.6431 | 0.0033 | Y |
| 270 | 11 | 17 | 0 | 0.1448 | 0.0112 | 1.7689 | 0.1835 | 3.7932 | 0.0515 | Y |
| 271 | 11 | 18 | 0 | 0.1239 | 0.0090 | 1.2746 | 0.2589 | 3.0414 | 0.0812 | Y |
| 272 | 11 | 19 | 0 | 0.1673 | 0.0097 | 1.8508 | 0.1737 | 3.2616 | 0.0709 | Y |
| 273 | 11 | 20 | 0 | 0.2189 | 0.0294 | 4.9785 | 0.0257 | 9.9332 | 0.0016 | Y |
| 274 | 11 | 21 | 0 | 0.1197 | 0.0100 | 1.0261 | 0.3111 | 3.3787 | 0.0661 | Y |
| 275 | 11 | 22 | 0 | 0.4354 | 0.0100 | 2.3363 | 0.1264 | 3.3686 | 0.0665 | Y |
| 276 | 11 | 23 | 0 | 0.0777 | 0.0039 | 0.7216 | 0.3956 | 1.3097 | 0.2524 | Y |
| 277 | 11 | 24 | 0 | 0.0092 | 0.0000 | -0.0035 | 1.0000 | 0.0129 | 0.9097 | Y |
| 278 | 11 | 25 | 0 | 0.0344 | 0.0004 | 0.0957 | 0.7571 | 0.1503 | 0.6983 | Y |
| 279 | 11 | 26 | 0 | 0.3603 | 0.0239 | 5.9772 | 0.0145 | 8.0864 | 0.0045 | Y |
| 280 | 11 | 27 | 0 | 0.2040 | 0.0170 | 3.4068 | 0.0649 | 5.7342 | 0.0166 | Y |
| 281 | 11 | 28 | 0 | 0.0142 | 0.0001 | 0.0361 | 0.8494 | 0.0407 | 0.8402 | Y |
| 282 | 11 | 29 | 0 | 0.1405 | 0.0065 | 1.7115 | 0.1908 | 2.1948 | 0.1385 | Y |
| 283 | 12 | 13 | 0 | 0.1617 | 0.0081 | 1.1659 | 0.2802 | 2.7321 | 0.0984 | Y |
| 284 | 12 | 14 | 0 | 0.0661 | 0.0025 | 0.5979 | 0.4394 | 0.8505 | 0.3564 | Y |
| 285 | 12 | 15 | 0 | 0.0260 | 0.0006 | 0.0135 | 0.9075 | 0.2063 | 0.6497 | Y |
| 286 | 12 | 16 | 0 | 0.0681 | 0.0025 | 0.1285 | 0.7200 | 0.8296 | 0.3624 | Y |
| 287 | 12 | 17 | 0 | 0.1368 | 0.0070 | 1.4895 | 0.2223 | 2.3497 | 0.1253 | Y |
| 288 | 12 | 18 | 0 | 0.0003 | 0.0000 | -0.0007 | 1.0000 | 0.0000 | 0.9987 | Y |
| 289 | 12 | 19 | 0 | 0.0680 | 0.0027 | 0.3788 | 0.5383 | 0.9013 | 0.3424 | Y |
| 290 | 12 | 20 | 0 | 0.1001 | 0.0030 | 0.6553 | 0.4182 | 1.0043 | 0.3163 | Y |
| 291 | 12 | 21 | 0 | 0.1158 | 0.0093 | 1.0777 | 0.2992 | 3.1441 | 0.0762 | Y |
| 292 | 12 | 22 | 0 | 0.1708 | 0.0077 | 1.7494 | 0.1860 | 2.6083 | 0.1063 | Y |
| 293 | 12 | 23 | 0 | 0.0833 | 0.0021 | 0.6364 | 0.4250 | 0.7243 | 0.3948 | Y |
| 294 | 12 | 24 | 0 | 0.0125 | 0.0002 | -0.0225 | 1.0000 | 0.0499 | 0.8233 | Y |
| 295 | 12 | 25 | 0 | 0.1546 | 0.0185 | 3.0328 | 0.0816 | 6.2613 | 0.0123 | Y |
| 296 | 12 | 26 | 0 | 0.0782 | 0.0023 | 0.4323 | 0.5109 | 0.7876 | 0.3748 | Y |
| 297 | 12 | 27 | 0 | 0.1034 | 0.0052 | 1.1712 | 0.2792 | 1.7605 | 0.1846 | Y |
| 298 | 12 | 28 | 0 | 0.0537 | 0.0024 | 0.6125 | 0.4338 | 0.7941 | 0.3729 | Y |
| 299 | 12 | 29 | 0 | 0.0145 | 0.0001 | 0.0655 | 0.7980 | 0.0483 | 0.8261 | Y |
| 300 | 13 | 14 | 0 | 0.1638 | 0.0116 | 2.1686 | 0.1409 | 3.9312 | 0.0474 | Y |
| 301 | 13 | 15 | 0 | 0.0665 | 0.0012 | -0.0647 | 1.0000 | 0.4175 | 0.5182 | Y |
| 302 | 13 | 16 | 0 | 0.0047 | 0.0000 | -0.0109 | 1.0000 | 0.0044 | 0.9469 | Y |
| 303 | 13 | 17 | 0 | 0.2350 | 0.0154 | 2.6978 | 0.1005 | 5.2195 | 0.0223 | Y |
| 304 | 13 | 18 | 0 | 0.1156 | 0.0012 | 0.2326 | 0.6296 | 0.3963 | 0.5290 | Y |
| 305 | 13 | 19 | 0 | 0.0822 | 0.0029 | 0.5046 | 0.4775 | 0.9900 | 0.3197 | Y |
| 306 | 13 | 20 | 0 | 0.0860 | 0.0029 | 0.4324 | 0.5108 | 0.9855 | 0.3209 | Y |
| 307 | 13 | 21 | 0 | 0.1160 | 0.0029 | 0.4456 | 0.5044 | 0.9745 | 0.3236 | Y |
| 308 | 13 | 22 | 0 | 0.0987 | 0.0034 | 0.7665 | 0.3813 | 1.1566 | 0.2822 | Y |
| 309 | 13 | 23 | 0 | 0.1581 | 0.0058 | 1.2958 | 0.2550 | 1.9676 | 0.1607 | Y |
| 310 | 13 | 24 | 0 | 0.0789 | 0.0021 | 0.7379 | 0.3903 | 0.6925 | 0.4053 | Y |
| 311 | 13 | 25 | 0 | 0.2662 | 0.0283 | 4.8279 | 0.0280 | 9.5597 | 0.0020 | Y |
| 312 | 13 | 26 | 0 | 0.0321 | 0.0003 | 0.1108 | 0.7392 | 0.1000 | 0.7519 | Y |
| 313 | 13 | 27 | 0 | 0.0043 | 0.0000 | 0.0125 | 0.9108 | 0.0040 | 0.9497 | Y |
| 314 | 13 | 28 | 0 | 0.0787 | 0.0016 | 0.0886 | 0.7660 | 0.5281 | 0.4674 | Y |
| 315 | 13 | 29 | 0 | 0.1095 | 0.0061 | 1.1053 | 0.2931 | 2.0754 | 0.1497 | Y |
| 316 | 14 | 15 | 0 | 0.0273 | 0.0005 | -0.0288 | 1.0000 | 0.1629 | 0.6865 | Y |
| 317 | 14 | 16 | 0 | 0.0997 | 0.0074 | 0.9894 | 0.3199 | 2.4954 | 0.1142 | Y |
| 318 | 14 | 17 | 0 | 0.1728 | 0.0193 | 3.2256 | 0.0725 | 6.5081 | 0.0107 | Y |
| 319 | 14 | 18 | 0 | 0.0057 | 0.0000 | 0.0021 | 0.9634 | 0.0018 | 0.9659 | Y |
| 320 | 14 | 19 | 0 | 0.0865 | 0.0060 | 1.0704 | 0.3009 | 2.0425 | 0.1530 | Y |
| 321 | 14 | 20 | 0 | 0.1188 | 0.0024 | 0.6388 | 0.4241 | 0.8150 | 0.3667 | Y |
| 322 | 14 | 21 | 0 | 0.0059 | 0.0000 | -0.0287 | 1.0000 | 0.0057 | 0.9397 | Y |
| 323 | 14 | 22 | 0 | 0.1468 | 0.0033 | 0.7530 | 0.3855 | 1.1097 | 0.2922 | Y |
| 324 | 14 | 23 | 0 | 0.0982 | 0.0052 | 0.6621 | 0.4158 | 1.7507 | 0.1858 | Y |
| 325 | 14 | 24 | 0 | 0.0404 | 0.0010 | 0.2066 | 0.6495 | 0.3382 | 0.5609 | Y |
| 326 | 14 | 25 | 0 | 0.1269 | 0.0148 | 2.6914 | 0.1009 | 5.0094 | 0.0252 | Y |
| 327 | 14 | 26 | 0 | 0.0319 | 0.0005 | 0.2181 | 0.6405 | 0.1837 | 0.6682 | Y |
| 328 | 14 | 27 | 0 | 0.0918 | 0.0071 | 1.6024 | 0.2056 | 2.4097 | 0.1206 | Y |
| 329 | 14 | 28 | 0 | 0.0048 | 0.0000 | -0.0043 | 1.0000 | 0.0037 | 0.9516 | Y |
| 330 | 14 | 29 | 0 | 0.0893 | 0.0068 | 0.9442 | 0.3312 | 2.2820 | 0.1309 | Y |
| 331 | 15 | 16 | 0 | 0.0693 | 0.0023 | 0.2446 | 0.6209 | 0.7772 | 0.3780 | Y |
| 332 | 15 | 17 | 0 | 0.0028 | 0.0000 | 0.0108 | 0.9173 | 0.0027 | 0.9586 | Y |
| 333 | 15 | 18 | 0 | 0.0898 | 0.0025 | 0.6564 | 0.4178 | 0.8560 | 0.3549 | Y |
| 334 | 15 | 19 | 0 | 0.0662 | 0.0028 | 0.5241 | 0.4691 | 0.9538 | 0.3288 | Y |
| 335 | 15 | 20 | 0 | 0.0471 | 0.0007 | 0.2079 | 0.6484 | 0.2462 | 0.6198 | Y |
| 336 | 15 | 21 | 0 | 0.0041 | 0.0000 | 0.0250 | 0.8743 | 0.0043 | 0.9477 | Y |
| 337 | 15 | 22 | 0 | 0.0531 | 0.0003 | 0.0412 | 0.8392 | 0.0937 | 0.7596 | Y |
| 338 | 15 | 23 | 0 | 0.0484 | 0.0020 | 0.4525 | 0.5011 | 0.6595 | 0.4167 | Y |
| 339 | 15 | 24 | 0 | 0.1793 | 0.0127 | 1.6341 | 0.2011 | 4.2933 | 0.0383 | Y |
| 340 | 15 | 25 | 0 | 0.0020 | 0.0000 | 0.0012 | 0.9724 | 0.0007 | 0.9795 | Y |
| 341 | 15 | 26 | 0 | 0.0726 | 0.0051 | 1.3274 | 0.2493 | 1.7381 | 0.1874 | Y |
| 342 | 15 | 27 | 0 | 0.2239 | 0.0221 | 4.5741 | 0.0325 | 7.4635 | 0.0063 | Y |
| 343 | 15 | 28 | 0 | 0.1014 | 0.0031 | 0.3365 | 0.5619 | 1.0527 | 0.3049 | Y |
| 344 | 15 | 29 | 0 | 0.0241 | 0.0004 | -0.0051 | 1.0000 | 0.1204 | 0.7286 | Y |
| 345 | 16 | 17 | 0 | 0.0917 | 0.0040 | 0.0757 | 0.7832 | 1.3629 | 0.2430 | Y |
| 346 | 16 | 18 | 0 | 0.2641 | 0.0105 | 2.5232 | 0.1122 | 3.5458 | 0.0597 | Y |
| 347 | 16 | 19 | 0 | 0.0024 | 0.0000 | 0.0063 | 0.9368 | 0.0014 | 0.9699 | Y |
| 348 | 16 | 20 | 0 | 0.2517 | 0.0100 | 1.9492 | 0.1627 | 3.3676 | 0.0665 | Y |
| 349 | 16 | 21 | 0 | 0.0564 | 0.0017 | 0.6241 | 0.4295 | 0.5777 | 0.4472 | Y |
| 350 | 16 | 22 | 0 | 0.0315 | 0.0002 | 0.0180 | 0.8934 | 0.0687 | 0.7933 | Y |
| 351 | 16 | 23 | 0 | 0.0063 | 0.0000 | 0.0249 | 0.8746 | 0.0077 | 0.9301 | Y |
| 352 | 16 | 24 | 0 | 0.0138 | 0.0002 | -0.0217 | 1.0000 | 0.0530 | 0.8179 | Y |
| 353 | 16 | 25 | 0 | 0.0167 | 0.0003 | 0.1333 | 0.7151 | 0.0940 | 0.7592 | Y |
| 354 | 16 | 26 | 0 | 0.0525 | 0.0020 | 0.2216 | 0.6379 | 0.6689 | 0.4134 | Y |
| 355 | 16 | 27 | 0 | 0.0873 | 0.0070 | 0.9271 | 0.3356 | 2.3701 | 0.1237 | Y |
| 356 | 16 | 28 | 0 | 0.0200 | 0.0002 | -0.0211 | 1.0000 | 0.0584 | 0.8090 | Y |
| 357 | 16 | 29 | 0 | 0.0413 | 0.0015 | -0.1158 | 1.0000 | 0.5052 | 0.4772 | Y |
| 358 | 17 | 18 | 0 | 0.0741 | 0.0017 | 0.3860 | 0.5344 | 0.5826 | 0.4453 | Y |
| 359 | 17 | 19 | 0 | 0.1032 | 0.0069 | 1.5070 | 0.2196 | 2.3220 | 0.1276 | Y |
| 360 | 17 | 20 | 0 | 0.0659 | 0.0014 | 0.4926 | 0.4828 | 0.4813 | 0.4878 | Y |
| 361 | 17 | 21 | 0 | 0.1523 | 0.0060 | 0.0570 | 0.8113 | 2.0194 | 0.1553 | Y |
| 362 | 17 | 22 | 0 | 0.2230 | 0.0146 | 3.2120 | 0.0731 | 4.9183 | 0.0266 | Y |
| 363 | 17 | 23 | 0 | 0.0929 | 0.0072 | 1.5414 | 0.2144 | 2.4306 | 0.1190 | Y |
| 364 | 17 | 24 | 0 | 0.0997 | 0.0084 | 1.6097 | 0.2045 | 2.8540 | 0.0912 | Y |
| 365 | 17 | 25 | 0 | 0.0230 | 0.0004 | 0.0066 | 0.9353 | 0.1247 | 0.7240 | Y |
| 366 | 17 | 26 | 0 | 0.1678 | 0.0097 | 1.1629 | 0.2809 | 3.2792 | 0.0702 | Y |
| 367 | 17 | 27 | 0 | 0.0933 | 0.0038 | 0.4997 | 0.4797 | 1.2971 | 0.2548 | Y |
| 368 | 17 | 28 | 0 | 0.0171 | 0.0003 | 0.0291 | 0.8646 | 0.0891 | 0.7653 | Y |
| 369 | 17 | 29 | 0 | 0.1060 | 0.0061 | 1.6954 | 0.1929 | 2.0721 | 0.1500 | Y |
| 370 | 18 | 19 | 0 | 0.2403 | 0.0117 | 2.0964 | 0.1477 | 3.9490 | 0.0469 | Y |
| 371 | 18 | 20 | 0 | 0.4363 | 0.0066 | 1.6311 | 0.2016 | 2.2238 | 0.1359 | Y |
| 372 | 18 | 21 | 0 | 0.1035 | 0.0009 | 0.0343 | 0.8531 | 0.2927 | 0.5885 | Y |
| 373 | 18 | 22 | 0 | 0.0170 | 0.0003 | 0.0675 | 0.7950 | 0.0913 | 0.7625 | Y |
| 374 | 18 | 23 | 0 | 0.0083 | 0.0000 | 0.0307 | 0.8609 | 0.0088 | 0.9252 | Y |
| 375 | 18 | 24 | 0 | 0.3187 | 0.0126 | 2.5374 | 0.1112 | 4.2555 | 0.0391 | Y |
| 376 | 18 | 25 | 0 | 0.0317 | 0.0002 | 0.0529 | 0.8181 | 0.0747 | 0.7846 | Y |
| 377 | 18 | 26 | 0 | 0.1329 | 0.0019 | 0.3398 | 0.5599 | 0.6450 | 0.4219 | Y |
| 378 | 18 | 27 | 0 | 0.0622 | 0.0005 | 0.0396 | 0.8422 | 0.1806 | 0.6709 | Y |
| 379 | 18 | 28 | 0 | 0.3827 | 0.0139 | 2.9917 | 0.0837 | 4.7037 | 0.0301 | Y |
| 380 | 18 | 29 | 0 | 0.2150 | 0.0089 | 1.3867 | 0.2390 | 3.0142 | 0.0825 | Y |
| 381 | 19 | 20 | 0 | 0.2280 | 0.0110 | 2.0126 | 0.1560 | 3.7160 | 0.0539 | Y |
| 382 | 19 | 21 | 0 | 0.0781 | 0.0024 | 0.3431 | 0.5581 | 0.8243 | 0.3639 | Y |
| 383 | 19 | 22 | 0 | 0.3123 | 0.0149 | 3.0311 | 0.0817 | 5.0230 | 0.0250 | Y |
| 384 | 19 | 23 | 0 | 0.0427 | 0.0010 | 0.1808 | 0.6707 | 0.3312 | 0.5650 | Y |
| 385 | 19 | 24 | 0 | 0.0109 | 0.0001 | 0.0111 | 0.9160 | 0.0246 | 0.8755 | Y |
| 386 | 19 | 25 | 0 | 0.0518 | 0.0020 | 0.3976 | 0.5284 | 0.6742 | 0.4116 | Y |
| 387 | 19 | 26 | 0 | 0.0774 | 0.0032 | 0.5978 | 0.4394 | 1.0828 | 0.2981 | Y |
| 388 | 19 | 27 | 0 | 0.0163 | 0.0002 | 0.0087 | 0.9256 | 0.0757 | 0.7832 | Y |
| 389 | 19 | 28 | 0 | 0.1506 | 0.0132 | 2.2569 | 0.1330 | 4.4576 | 0.0348 | Y |
| 390 | 19 | 29 | 0 | 0.0323 | 0.0009 | 0.2396 | 0.6245 | 0.2995 | 0.5842 | Y |
| 391 | 20 | 21 | 0 | 0.4917 | 0.0204 | 1.5565 | 0.2122 | 6.9061 | 0.0086 | Y |
| 392 | 20 | 22 | 0 | 0.0414 | 0.0015 | 0.3521 | 0.5529 | 0.5166 | 0.4723 | Y |
| 393 | 20 | 23 | 0 | 0.4305 | 0.0170 | 2.2790 | 0.1311 | 5.7468 | 0.0165 | Y |
| 394 | 20 | 24 | 0 | 0.0021 | 0.0000 | -0.0010 | 1.0000 | 0.0002 | 0.9890 | Y |
| 395 | 20 | 25 | 0 | 0.2444 | 0.0094 | 1.1175 | 0.2905 | 3.1751 | 0.0748 | Y |
| 396 | 20 | 26 | 0 | 0.1513 | 0.0026 | 0.4243 | 0.5148 | 0.8748 | 0.3496 | Y |
| 397 | 20 | 27 | 0 | 0.0840 | 0.0010 | 0.2479 | 0.6186 | 0.3450 | 0.5569 | Y |
| 398 | 20 | 28 | 0 | 0.4076 | 0.0604 | 10.6307 | 0.0011 | 20.4300 | 0.0000 | Y |
| 399 | 20 | 29 | 0 | 0.0081 | 0.0000 | 0.0244 | 0.8758 | 0.0040 | 0.9499 | Y |
| 400 | 21 | 22 | 0 | 0.1290 | 0.0013 | 0.2446 | 0.6209 | 0.4240 | 0.5150 | Y |
| 401 | 21 | 23 | 0 | 0.1086 | 0.0109 | 2.8486 | 0.0915 | 3.6744 | 0.0553 | Y |
| 402 | 21 | 24 | 0 | 0.1550 | 0.0073 | 1.2849 | 0.2570 | 2.4605 | 0.1167 | Y |
| 403 | 21 | 25 | 0 | 0.0086 | 0.0000 | -0.0224 | 1.0000 | 0.0092 | 0.9235 | Y |
| 404 | 21 | 26 | 0 | 0.0551 | 0.0023 | 0.6327 | 0.4264 | 0.7682 | 0.3808 | Y |
| 405 | 21 | 27 | 0 | 0.0266 | 0.0002 | 0.0924 | 0.7612 | 0.0810 | 0.7760 | Y |
| 406 | 21 | 28 | 0 | 0.1915 | 0.0085 | 1.9752 | 0.1599 | 2.8777 | 0.0898 | Y |
| 407 | 21 | 29 | 0 | 0.1587 | 0.0106 | 3.0968 | 0.0785 | 3.5660 | 0.0590 | Y |
| 408 | 22 | 23 | 0 | 0.0769 | 0.0021 | 0.4627 | 0.4964 | 0.7015 | 0.4023 | Y |
| 409 | 22 | 24 | 0 | 0.0175 | 0.0001 | 0.0011 | 0.9741 | 0.0256 | 0.8728 | Y |
| 410 | 22 | 25 | 0 | 0.0167 | 0.0000 | 0.0048 | 0.9450 | 0.0133 | 0.9083 | Y |
| 411 | 22 | 26 | 0 | 0.3374 | 0.0115 | 2.1846 | 0.1394 | 3.8775 | 0.0489 | Y |
| 412 | 22 | 27 | 0 | 0.0975 | 0.0021 | 0.4904 | 0.4838 | 0.7163 | 0.3974 | Y |
| 413 | 22 | 28 | 0 | 0.0761 | 0.0019 | 0.4443 | 0.5051 | 0.6344 | 0.4257 | Y |
| 414 | 22 | 29 | 0 | 0.1524 | 0.0037 | 0.8358 | 0.3606 | 1.2551 | 0.2626 | Y |
| 415 | 23 | 24 | 0 | 0.1547 | 0.0079 | 1.4672 | 0.2258 | 2.6596 | 0.1029 | Y |
| 416 | 23 | 25 | 0 | 0.0053 | 0.0000 | -0.0076 | 1.0000 | 0.0037 | 0.9513 | Y |
| 417 | 23 | 26 | 0 | 0.2696 | 0.0208 | 3.4345 | 0.0639 | 7.0426 | 0.0080 | Y |
| 418 | 23 | 27 | 0 | 0.0988 | 0.0062 | 1.4304 | 0.2317 | 2.0922 | 0.1481 | Y |
| 419 | 23 | 28 | 0 | 0.0676 | 0.0012 | 0.2340 | 0.6286 | 0.3890 | 0.5328 | Y |
| 420 | 23 | 29 | 0 | 0.0466 | 0.0011 | -0.0047 | 1.0000 | 0.3757 | 0.5399 | Y |
| 421 | 24 | 25 | 0 | 0.1582 | 0.0206 | 3.6623 | 0.0557 | 6.9668 | 0.0083 | Y |
| 422 | 24 | 26 | 0 | 0.0648 | 0.0037 | 0.5962 | 0.4400 | 1.2367 | 0.2661 | Y |
| 423 | 24 | 27 | 0 | 0.0856 | 0.0066 | 1.0689 | 0.3012 | 2.2210 | 0.1361 | Y |
| 424 | 24 | 28 | 0 | 0.0463 | 0.0017 | 0.3280 | 0.5669 | 0.5560 | 0.4559 | Y |
| 425 | 24 | 29 | 0 | 0.0307 | 0.0007 | 0.1097 | 0.7405 | 0.2300 | 0.6315 | Y |
| 426 | 25 | 26 | 0 | 0.1745 | 0.0219 | 3.5040 | 0.0612 | 7.3960 | 0.0065 | Y |
| 427 | 25 | 27 | 0 | 0.0845 | 0.0066 | 0.9303 | 0.3348 | 2.2179 | 0.1364 | Y |
| 428 | 25 | 28 | 0 | 0.0400 | 0.0007 | -0.1513 | 1.0000 | 0.2336 | 0.6289 | Y |
| 429 | 25 | 29 | 0 | 0.0495 | 0.0022 | 0.0609 | 0.8051 | 0.7271 | 0.3938 | Y |
| 430 | 26 | 27 | 0 | 0.0187 | 0.0003 | 0.1022 | 0.7492 | 0.0924 | 0.7611 | Y |
| 431 | 26 | 28 | 0 | 0.0389 | 0.0013 | 0.3332 | 0.5638 | 0.4486 | 0.5030 | Y |
| 432 | 26 | 29 | 0 | 0.0104 | 0.0001 | -0.0202 | 1.0000 | 0.0232 | 0.8790 | Y |
| 433 | 27 | 28 | 0 | 0.0101 | 0.0000 | 0.0266 | 0.8703 | 0.0137 | 0.9069 | Y |
| 434 | 27 | 29 | 0 | 0.0712 | 0.0041 | 0.9162 | 0.3385 | 1.3805 | 0.2400 | Y |
| 435 | 28 | 29 | 0 | 0.0044 | 0.0000 | -0.0327 | 1.0000 | 0.0032 | 0.9550 | Y |

Marker 1: HLD 77; marker2: HLD 45; marker 3: HLD131; marker 4: HLD 70: marker 5: HLD 6; marker 6: HLD111; marker 7: HLD 58; marker 8: HLD 56; marker 9: HLD 118;

Marker 10: HLD 92; marker 11: HLD 93; marker 12: HLD 99; marker 13: HLD 88; marker 14: HLD 101; marker 15: HLD 67; marker 16: HLD 83; marker 17: HLD 114; marker 18: HLD48;

Marker 19: HLD 124; marker 20: HLD 122; marker 21: HLD 125; marker 22: HLD 64; marker 23: HLD 81; marker 24: HLD 136; marker 25: HLD 133; marker 26: HLD 97; marker 27: HLD 40;

Marker 28: HLD 128; marker 29: HLD 39; marker 30: HLD 84
