# Supplementary material for: Population Genetic Diversity and Clustering Analysis for Chinese Dongxiang Group With 30 Autosomal InDel Loci Simultaneously Analyzed
Source: Front Genet. 2018 Aug 2;9:279. doi: 10.3389/fgene.2018.00279 (PMC6082941; doi:10.3389/fgene.2018.00279)
Supplement: TABLE S2 — P values of pairwise population differentiations between Dongxiang and the 30 reference populations. [file Table_2.DOCX]

| **Table 2. *P* values of pairwise population differentiations between Dongxiang and the 30 reference populations.** | | | | | | | | | | | |  |  |  |  |  |  |  |  |  |  |  |  |  |  |  |  |  |  |  |  |
| --- | --- | --- | --- | --- | --- | --- | --- | --- | --- | --- | --- | --- | --- | --- | --- | --- | --- | --- | --- | --- | --- | --- | --- | --- | --- | --- | --- | --- | --- | --- | --- |
| **Populations** | | **HLD77** | **HLD45** | **HLD131** | **HLD70** | **HLD6** | **HLD111** | **HLD58** | **HLD56** | **HLD118** | **HLD92** | **HLD93** | **HLD99** | **HLD88** | **HLD101** | **HLD67** | **HLD83** | **HLD114** | **HLD48** | **HLD124** | **HLD122** | **HLD125** | **HLD64** | **HLD81** | **HLD136** | **HLD133** | **HLD97** | **HLD40** | **HLD128** | **HLD39** | **HLD84** |
| **Cape Colored** | | **0.1056** | **0.0000** | **0.0108** | **0.0000** | **0.0049** | **0.0000** | **0.4858** | **0.3715** | **0.0000** | **0.4467** | **0.4946** | **0.0010** | **0.0401** | **0.0000** | **0.0450** | **1.0000** | **0.0000** | **0.0000** | **0.0029** | **1.0000** | **1.0000** | **1.0000** | **0.0020** | **1.0000** | **1.0000** | **0.4350** | **0.0000** | **0.0010** | **0.0000** | **0.0000** |
| **Xhosa** | | **0.0000** | **0.0000** | **0.0000** | **0.0000** | **0.0010** | **0.0000** | **0.0000** | **1.0000** | **0.0000** | **1.0000** | **0.3939** | **0.0000** | **0.0000** | **0.0000** | **0.0772** | **0.0000** | **0.0000** | **0.0000** | **0.0000** | **0.5699** | **0.0000** | **1.0000** | **0.0000** | **0.0000** | **1.0000** | **0.5523** | **0.0000** | **0.0000** | **0.0000** | **0.0000** |
| **Zulu** | | **0.0147** | **0.0000** | **0.0000** | **0.0000** | **0.0000** | **0.0010** | **0.0000** | **0.0626** | **0.0000** | **0.7693** | **1.0000** | **0.0000** | **0.0000** | **0.0000** | **0.0049** | **0.0000** | **0.0000** | **0.0000** | **0.0000** | **1.0000** | **0.0000** | **0.4018** | **0.0000** | **0.0000** | **1.0000** | **1.0000** | **0.0000** | **0.0000** | **0.0000** | **0.0000** |
| **Chihuahua Mexican** | | **1.0000** | **0.3333** | **0.0254** | **0.3763** | **0.0000** | **0.0000** | **0.0068** | **0.0020** | **0.0000** | **0.0782** | **1.0000** | **0.0000** | **1.0000** | **0.2522** | **1.0000** | **0.0068** | **0.0029** | **0.0000** | **0.7546** | **0.9218** | **0.0000** | **0.0244** | **0.0020** | **0.3666** | **0.0166** | **0.4301** | **0.0000** | **0.0508** | **0.0049** | **0.0010** |
| **Jalisco Mexican** | | **1.0000** | **1.0000** | **1.0000** | **0.1222** | **0.1975** | **0.0000** | **0.1261** | **0.0274** | **0.0000** | **0.5885** | **1.0000** | **0.0000** | **1.0000** | **0.8162** | **1.0000** | **0.2014** | **0.0020** | **0.0000** | **0.4868** | **1.0000** | **0.0117** | **0.1134** | **0.0010** | **0.0479** | **0.0411** | **0.7136** | **0.0049** | **0.3519** | **0.0948** | **0.0000** |
| **Mexico Mexican** | | **0.1623** | **0.6774** | **0.4594** | **0.0000** | **1.0000** | **0.0000** | **0.0029** | **0.0000** | **0.0000** | **0.2669** | **1.0000** | **0.0000** | **0.0156** | **0.0323** | **0.4360** | **0.0000** | **0.0039** | **0.0000** | **0.4721** | **1.0000** | **0.0000** | **0.0156** | **0.0010** | **1.0000** | **1.0000** | **0.0332** | **0.0655** | **1.0000** | **0.0000** | **0.0000** |
| **Veracruz Mexican** | | **0.1730** | **0.2532** | **0.2160** | **0.0127** | **0.5464** | **0.0000** | **0.0606** | **0.4985** | **0.0000** | **0.1916** | **0.1007** | **0.0000** | **0.0547** | **1.0000** | **1.0000** | **0.0000** | **0.0499** | **0.0000** | **1.0000** | **1.0000** | **0.0000** | **0.5992** | **0.0323** | **0.4497** | **0.1124** | **0.1144** | **0.0655** | **0.8935** | **0.0010** | **0.0029** |
| **Yucatan Mexican** | | **1.0000** | **1.0000** | **0.8328** | **0.0000** | **0.1994** | **0.0000** | **0.0000** | **0.0000** | **0.0000** | **0.0000** | **0.5777** | **0.0000** | **0.0313** | **1.0000** | **1.0000** | **0.0010** | **0.0968** | **0.0000** | **1.0000** | **0.0567** | **0.0000** | **0.0547** | **1.0000** | **0.3734** | **0.1427** | **0.3324** | **0.1828** | **0.1994** | **0.0000** | **0.0000** |
| **Amerindian Mexican** | | **1.0000** | **0.0655** | **0.5181** | **0.0059** | **0.0000** | **0.3343** | **0.0000** | **0.1241** | **0.0000** | **0.0342** | **0.0733** | **0.0000** | **1.0000** | **0.3148** | **1.0000** | **0.0000** | **0.0010** | **0.0000** | **1.0000** | **0.1896** | **0.0000** | **0.0000** | **0.2444** | **0.0000** | **0.5582** | **1.0000** | **0.2346** | **0.2884** | **0.0000** | **0.0000** |
| **Dane** | | **0.0068** | **0.0000** | **0.0763** | **0.2180** | **1.0000** | **0.0000** | **0.0010** | **0.1652** | **0.0000** | **0.0332** | **0.3167** | **0.0000** | **0.3529** | **0.0411** | **1.0000** | **0.0000** | **0.1965** | **0.0000** | **0.5943** | **0.3822** | **0.0391** | **0.0000** | **0.0000** | **1.0000** | **0.0000** | **0.0000** | **0.0010** | **0.3265** | **0.0156** | **0.1750** |
| **Hungarian** | | **1.0000** | **0.0000** | **0.0000** | **0.7048** | **1.0000** | **0.0000** | **0.0010** | **0.0039** | **0.0000** | **0.2913** | **0.8768** | **0.0000** | **0.2894** | **1.0000** | **1.0000** | **0.0811** | **0.3803** | **0.0450** | **0.3128** | **0.0000** | **0.0127** | **0.0000** | **0.0000** | **0.5865** | **0.0000** | **0.0000** | **0.0000** | **0.0059** | **0.0000** | **0.0000** |
| **Basque** | | **0.0381** | **0.0000** | **0.5865** | **0.1623** | **0.5132** | **0.0000** | **0.0000** | **1.0000** | **0.0000** | **0.5083** | **0.3441** | **0.0000** | **0.0284** | **0.7165** | **0.0841** | **0.0029** | **1.0000** | **0.0176** | **0.0929** | **1.0000** | **1.0000** | **0.0000** | **0.0000** | **0.0000** | **0.0000** | **0.0000** | **0.0000** | **0.0117** | **0.1349** | **0.0841** |
| **Central Spanish** | | **0.4184** | **0.0029** | **0.2884** | **0.5660** | **1.0000** | **0.0000** | **0.0039** | **0.0010** | **0.0000** | **0.0156** | **0.6100** | **0.0000** | **0.0469** | **0.6970** | **0.3167** | **0.0137** | **0.0000** | **0.0029** | **0.3812** | **0.1564** | **1.0000** | **0.0000** | **0.0000** | **0.5904** | **0.0000** | **0.0039** | **0.0000** | **0.1525** | **0.0000** | **0.1896** |
| **Kazak** | | **1.0000** | **0.4096** | **1.0000** | **1.0000** | **0.0675** | **0.0000** | **1.0000** | **1.0000** | **0.0000** | **0.0000** | **0.4546** | **0.0000** | **0.2454** | **0.1672** | **1.0000** | **0.6422** | **0.0059** | **0.4477** | **1.0000** | **0.6149** | **1.0000** | **0.0068** | **0.1574** | **1.0000** | **0.0147** | **1.0000** | **0.0596** | **0.7713** | **1.0000** | **1.0000** |
| **Uyghur** | | **0.8544** | **0.1085** | **0.2180** | **1.0000** | **1.0000** | **0.0000** | **1.0000** | **0.0831** | **0.0000** | **0.0049** | **1.0000** | **0.0000** | **0.1623** | **1.0000** | **0.3412** | **1.0000** | **1.0000** | **1.0000** | **0.4458** | **0.0020** | **0.6354** | **0.0244** | **0.0020** | **0.5670** | **0.0068** | **0.8495** | **0.0587** | **0.1672** | **0.0059** | **0.2727** |
| **Hui** | | **1.0000** | **0.1554** | **0.0821** | **1.0000** | **1.0000** | **0.4966** | **1.0000** | **0.5562** | **0.7331** | **0.0078** | **1.0000** | **1.0000** | **0.5435** | **0.2405** | **0.0010** | **0.0919** | **0.2571** | **1.0000** | **0.6980** | **0.0156** | **0.0332** | **0.8153** | **0.0235** | **0.5064** | **0.0899** | **0.5611** | **0.0000** | **0.0000** | **0.0000** | **1.0000** |
| **Xibe** | | **0.2004** | **0.3236** | **0.0010** | **0.2620** | **0.2239** | **0.1779** | **0.0137** | **1.0000** | **0.0196** | **0.0020** | **0.0411** | **1.0000** | **0.2317** | **1.0000** | **0.0000** | **1.0000** | **1.0000** | **1.0000** | **1.0000** | **0.1896** | **0.2942** | **0.3724** | **0.0137** | **0.7243** | **0.0821** | **1.0000** | **1.0000** | **1.0000** | **0.0772** | **0.0000** |
| **Yi** | | **0.4819** | **0.0577** | **1.0000** | **0.1896** | **0.4917** | **0.1975** | **0.0606** | **0.8016** | **0.0127** | **0.5758** | **0.4751** | **0.1642** | **0.6012** | **1.0000** | **0.0049** | **1.0000** | **1.0000** | **1.0000** | **0.7155** | **0.0029** | **0.0284** | **0.1222** | **0.1388** | **1.0000** | **0.5083** | **0.2121** | **0.2248** | **0.0362** | **0.0518** | **0.5533** |
| **Zhuang** | | **0.0684** | **0.5494** | **0.0010** | **1.0000** | **1.0000** | **1.0000** | **0.5103** | **0.5083** | **0.4477** | **0.0068** | **0.1574** | **0.4184** | **1.0000** | **0.8133** | **0.0010** | **1.0000** | **0.2962** | **0.6667** | **0.5582** | **0.0000** | **1.0000** | **0.0332** | **0.5240** | **0.4330** | **0.0147** | **1.0000** | **1.0000** | **1.0000** | **0.0538** | **0.0039** |
| **Dong** | | **0.1026** | **1.0000** | **0.0068** | **1.0000** | **0.2542** | **1.0000** | **0.0401** | **0.0616** | **0.0029** | **0.0938** | **0.2454** | **1.0000** | **0.1437** | **0.0645** | **0.0000** | **0.2033** | **0.0313** | **1.0000** | **0.1457** | **0.0450** | **0.5425** | **0.0147** | **0.5826** | **1.0000** | **1.0000** | **0.0166** | **0.0440** | **0.0156** | **0.0010** | **0.0000** |
| **Tujia** | | **1.0000** | **1.0000** | **0.0088** | **1.0000** | **1.0000** | **0.0049** | **1.0000** | **0.6471** | **0.0000** | **0.0186** | **1.0000** | **1.0000** | **0.9374** | **1.0000** | **0.0000** | **1.0000** | **0.0303** | **0.3157** | **1.0000** | **0.0049** | **0.0029** | **0.0215** | **0.0284** | **1.0000** | **1.0000** | **0.6276** | **0.2874** | **1.0000** | **0.0000** | **0.0127** |
| **Miao** | | **0.0811** | **0.2346** | **0.4467** | **1.0000** | **0.0156** | **1.0000** | **0.2151** | **0.3109** | **0.3597** | **1.0000** | **1.0000** | **0.2082** | **0.3529** | **0.0850** | **0.0000** | **1.0000** | **0.0098** | **1.0000** | **0.0068** | **0.0000** | **0.7967** | **0.0274** | **0.7889** | **0.0010** | **0.3646** | **1.0000** | **0.6276** | **0.0284** | **0.0313** | **0.0049** |
| **She** | | **1.0000** | **1.0000** | **0.0479** | **0.0948** | **0.0244** | **0.3304** | **1.0000** | **0.6031** | **1.0000** | **0.0919** | **0.0303** | **0.2033** | **0.4164** | **0.0391** | **0.0000** | **0.0117** | **0.2718** | **1.0000** | **0.0547** | **0.0000** | **0.2669** | **0.0313** | **0.6110** | **0.1075** | **0.5435** | **1.0000** | **0.0254** | **0.2151** | **0.0000** | **0.0000** |
| **Tibet Tibetan** | | **0.0606** | **0.2033** | **1.0000** | **0.3470** | **1.0000** | **0.0137** | **0.0166** | **0.0870** | **0.0342** | **0.0000** | **0.5259** | **0.0508** | **0.2063** | **1.0000** | **1.0000** | **0.0156** | **0.2835** | **0.1447** | **0.4184** | **1.0000** | **1.0000** | **0.0137** | **0.0117** | **0.2258** | **0.0254** | **0.0264** | **0.2170** | **0.0489** | **0.3480** | **1.0000** |
| **Qinghai Tibetan** | | **1.0000** | **1.0000** | **0.6931** | **1.0000** | **0.3451** | **0.0518** | **0.1134** | **0.1936** | **0.0342** | **0.1691** | **0.3099** | **1.0000** | **0.0049** | **1.0000** | **0.0264** | **0.5591** | **0.7742** | **1.0000** | **0.0000** | **0.7253** | **0.5826** | **0.0821** | **0.0225** | **0.4976** | **0.3656** | **0.4282** | **0.9257** | **0.1486** | **0.4428** | **1.0000** |
| **Chengdu Han** | | **1.0000** | **0.0147** | **0.0547** | **0.4272** | **1.0000** | **0.0313** | **1.0000** | **0.1720** | **0.0098** | **1.0000** | **0.2688** | **0.3245** | **0.0186** | **0.4731** | **0.0000** | **1.0000** | **1.0000** | **1.0000** | **0.2454** | **0.0860** | **0.4233** | **1.0000** | **0.4790** | **0.3324** | **0.2571** | **1.0000** | **0.4849** | **0.3949** | **0.0284** | **0.0020** |
| **Beijing Han** | | **0.5249** | **0.0156** | **0.0068** | **1.0000** | **1.0000** | **0.0088** | **0.2063** | **0.2121** | **0.0264** | **0.0059** | **0.1711** | **0.3324** | **0.9404** | **1.0000** | **0.0127** | **1.0000** | **1.0000** | **1.0000** | **1.0000** | **0.1916** | **0.0489** | **1.0000** | **0.0000** | **0.1173** | **0.8720** | **0.3539** | **0.1339** | **0.0176** | **0.0078** | **0.2796** |
| **Henan Han** | | **1.0000** | **0.0508** | **0.3245** | **0.2278** | **1.0000** | **0.0059** | **1.0000** | **0.1691** | **0.0000** | **0.1955** | **1.0000** | **0.1887** | **0.4731** | **1.0000** | **0.0000** | **0.5259** | **0.2024** | **1.0000** | **1.0000** | **0.0088** | **0.0000** | **0.4976** | **0.0020** | **0.6198** | **1.0000** | **1.0000** | **0.2366** | **0.3431** | **0.0010** | **0.0489** |
| **Shanghai Han** | | **0.8622** | **0.0782** | **0.0068** | **1.0000** | **1.0000** | **0.0000** | **1.0000** | **0.0763** | **0.0000** | **0.0127** | **1.0000** | **0.3607** | **1.0000** | **1.0000** | **0.0010** | **0.4526** | **0.2630** | **1.0000** | **0.1261** | **0.0010** | **0.0000** | **0.1867** | **0.0274** | **1.0000** | **1.0000** | **0.4917** | **0.0068** | **0.0772** | **0.0010** | **0.0176** |
| **Guangdong Han** | | **0.7810** | **0.2933** | **0.0059** | **1.0000** | **0.2854** | **0.0156** | **0.0313** | **0.0108** | **0.0020** | **0.0010** | **1.0000** | **0.1124** | **1.0000** | **1.0000** | **0.0000** | **0.6070** | **0.0420** | **1.0000** | **0.0215** | **0.0000** | **0.0528** | **0.1545** | **0.4282** | **0.2698** | **1.0000** | **1.0000** | **0.0166** | **0.5132** | **0.0000** | **0.0000** |
|  |  | |  |  |  |  |  |  |  |  |  |  |  |  |  |  |  |  |  |  |  |  |  |  |  |  |  |  |  |  |  |
