# Supplementary material for: Population Genetic Diversity and Clustering Analysis for Chinese Dongxiang Group With 30 Autosomal InDel Loci Simultaneously Analyzed
Source: Front Genet. 2018 Aug 2;9:279. doi: 10.3389/fgene.2018.00279 (PMC6082941; doi:10.3389/fgene.2018.00279)
Supplement: TABLE S3 — DA distance values for Chinese Dongxiang group and the 30 reference populations. [file Table_3.docx]

　Supplemental Table 3. *D_A_* distance values for Chinese Dongxiang group and the 30 reference populations.

| Populations | Dongxiang | Cape Colored | Xhosa | Zulu | Chihuahua Mexican | Jalisco Mexican | Mexico Mexican | Veracruz Mexican | Yucatan Mexican | Amerindian Mexican | Dane | Hungarian | Basque | Central Spanish | Kazak | Uyghur | Hui | Xibe | Yi | Zhuang | Dong | Tujia | Miao | She | Tibet Tibetan | Qinghai Tibetan | Chengdu Han | Beijing Han | Henan Han | Shanghai Han |
| --- | --- | --- | --- | --- | --- | --- | --- | --- | --- | --- | --- | --- | --- | --- | --- | --- | --- | --- | --- | --- | --- | --- | --- | --- | --- | --- | --- | --- | --- | --- |
| Cape Colored | 0.0159 |  |  |  |  |  |  |  |  |  |  |  |  |  |  |  |  |  |  |  |  |  |  |  |  |  |  |  |  |  |
| Xhosa | 0.0426 | 0.0116 |  |  |  |  |  |  |  |  |  |  |  |  |  |  |  |  |  |  |  |  |  |  |  |  |  |  |  |  |
| Zulu | 0.0480 | 0.0150 | 0.0017 |  |  |  |  |  |  |  |  |  |  |  |  |  |  |  |  |  |  |  |  |  |  |  |  |  |  |  |
| Chihuahua Mexican | 0.0182 | 0.0088 | 0.0258 | 0.0325 |  |  |  |  |  |  |  |  |  |  |  |  |  |  |  |  |  |  |  |  |  |  |  |  |  |  |
| Jalisco Mexican | 0.0169 | 0.0080 | 0.0249 | 0.0317 | 0.0012 |  |  |  |  |  |  |  |  |  |  |  |  |  |  |  |  |  |  |  |  |  |  |  |  |  |
| Mexico Mexican | 0.0224 | 0.0092 | 0.0235 | 0.0308 | 0.0047 | 0.0040 |  |  |  |  |  |  |  |  |  |  |  |  |  |  |  |  |  |  |  |  |  |  |  |  |
| Veracruz Mexican | 0.0189 | 0.0104 | 0.0276 | 0.0333 | 0.0036 | 0.0026 | 0.0033 |  |  |  |  |  |  |  |  |  |  |  |  |  |  |  |  |  |  |  |  |  |  |  |
| Yucatan Mexican | 0.0205 | 0.0121 | 0.0291 | 0.0360 | 0.0044 | 0.0046 | 0.0042 | 0.0032 |  |  |  |  |  |  |  |  |  |  |  |  |  |  |  |  |  |  |  |  |  |  |
| Amerindian Mexican | 0.0293 | 0.0209 | 0.0331 | 0.0401 | 0.0089 | 0.0090 | 0.0087 | 0.0065 | 0.0053 |  |  |  |  |  |  |  |  |  |  |  |  |  |  |  |  |  |  |  |  |  |
| Dane | 0.0171 | 0.0124 | 0.0371 | 0.0422 | 0.0093 | 0.0088 | 0.0174 | 0.0130 | 0.0167 | 0.0290 |  |  |  |  |  |  |  |  |  |  |  |  |  |  |  |  |  |  |  |  |
| Hungarian | 0.0168 | 0.0102 | 0.0335 | 0.0398 | 0.0092 | 0.0091 | 0.0155 | 0.0134 | 0.0175 | 0.0310 | 0.0026 |  |  |  |  |  |  |  |  |  |  |  |  |  |  |  |  |  |  |  |
| Basque | 0.0179 | 0.0142 | 0.0378 | 0.0445 | 0.0113 | 0.0106 | 0.0186 | 0.0159 | 0.0203 | 0.0312 | 0.0047 | 0.0046 |  |  |  |  |  |  |  |  |  |  |  |  |  |  |  |  |  |  |
| Central Spanish | 0.0173 | 0.0091 | 0.0294 | 0.0353 | 0.0080 | 0.0075 | 0.0137 | 0.0123 | 0.0154 | 0.0262 | 0.0029 | 0.0022 | 0.0033 |  |  |  |  |  |  |  |  |  |  |  |  |  |  |  |  |  |
| Kazak | 0.0047 | 0.0105 | 0.0352 | 0.0405 | 0.0090 | 0.0079 | 0.0137 | 0.0097 | 0.0123 | 0.0217 | 0.0089 | 0.0085 | 0.0111 | 0.0085 |  |  |  |  |  |  |  |  |  |  |  |  |  |  |  |  |
| Uyghur | 0.0047 | 0.0092 | 0.0347 | 0.0410 | 0.0085 | 0.0080 | 0.0137 | 0.0108 | 0.0129 | 0.0229 | 0.0071 | 0.0055 | 0.0090 | 0.0062 | 0.0019 |  |  |  |  |  |  |  |  |  |  |  |  |  |  |  |
| Hui | 0.0063 | 0.0175 | 0.0410 | 0.0459 | 0.0217 | 0.0212 | 0.0244 | 0.0203 | 0.0208 | 0.0288 | 0.0218 | 0.0212 | 0.0236 | 0.0203 | 0.0102 | 0.0098 |  |  |  |  |  |  |  |  |  |  |  |  |  |  |
| Xibe | 0.0024 | 0.0211 | 0.0481 | 0.0526 | 0.0231 | 0.0219 | 0.0269 | 0.0219 | 0.0240 | 0.0329 | 0.0220 | 0.0233 | 0.0236 | 0.0226 | 0.0068 | 0.0088 | 0.0075 |  |  |  |  |  |  |  |  |  |  |  |  |  |
| Yi | 0.0041 | 0.0222 | 0.0477 | 0.0513 | 0.0259 | 0.0246 | 0.0293 | 0.0245 | 0.0252 | 0.0345 | 0.0259 | 0.0274 | 0.0283 | 0.0266 | 0.0107 | 0.0124 | 0.0083 | 0.0022 |  |  |  |  |  |  |  |  |  |  |  |  |
| Zhuang | 0.0051 | 0.0249 | 0.0541 | 0.0588 | 0.0272 | 0.0262 | 0.0311 | 0.0271 | 0.0279 | 0.0373 | 0.0242 | 0.0264 | 0.0243 | 0.0245 | 0.0105 | 0.0120 | 0.0102 | 0.0039 | 0.0051 |  |  |  |  |  |  |  |  |  |  |  |
| Dong | 0.0086 | 0.0349 | 0.0656 | 0.0702 | 0.0383 | 0.0355 | 0.0422 | 0.0356 | 0.0373 | 0.0464 | 0.0337 | 0.0366 | 0.0358 | 0.0362 | 0.0166 | 0.0190 | 0.0146 | 0.0059 | 0.0064 | 0.0042 |  |  |  |  |  |  |  |  |  |  |
| Tujia | 0.0029 | 0.0252 | 0.0536 | 0.0582 | 0.0278 | 0.0262 | 0.0311 | 0.0263 | 0.0279 | 0.0360 | 0.0266 | 0.0282 | 0.0281 | 0.0277 | 0.0104 | 0.0117 | 0.0088 | 0.0015 | 0.0021 | 0.0026 | 0.0031 |  |  |  |  |  |  |  |  |  |
| Miao | 0.0065 | 0.0278 | 0.0598 | 0.0638 | 0.0314 | 0.0294 | 0.0345 | 0.0301 | 0.0304 | 0.0413 | 0.0284 | 0.0303 | 0.0320 | 0.0308 | 0.0131 | 0.0148 | 0.0114 | 0.0067 | 0.0056 | 0.0040 | 0.0039 | 0.0042 |  |  |  |  |  |  |  |  |
| She | 0.0045 | 0.0257 | 0.0562 | 0.0601 | 0.0274 | 0.0262 | 0.0306 | 0.0262 | 0.0278 | 0.0354 | 0.0266 | 0.0291 | 0.0288 | 0.0285 | 0.0112 | 0.0127 | 0.0107 | 0.0032 | 0.0039 | 0.0033 | 0.0039 | 0.0019 | 0.0036 |  |  |  |  |  |  |  |
| Tibet Tibetan | 0.0028 | 0.0202 | 0.0458 | 0.0517 | 0.0214 | 0.0196 | 0.0243 | 0.0206 | 0.0204 | 0.0276 | 0.0238 | 0.0237 | 0.0261 | 0.0236 | 0.0072 | 0.0087 | 0.0056 | 0.0034 | 0.0045 | 0.0065 | 0.0089 | 0.0035 | 0.0076 | 0.0059 |  |  |  |  |  |  |
| Qinghai Tibetan | 0.0021 | 0.0186 | 0.0432 | 0.0480 | 0.0219 | 0.0204 | 0.0247 | 0.0210 | 0.0217 | 0.0299 | 0.0232 | 0.0232 | 0.0256 | 0.0235 | 0.0075 | 0.0088 | 0.0052 | 0.0019 | 0.0027 | 0.0052 | 0.0071 | 0.0023 | 0.0061 | 0.0041 | 0.0012 |  |  |  |  |  |
| Chengdu Han | 0.0024 | 0.0234 | 0.0521 | 0.0564 | 0.0269 | 0.0256 | 0.0311 | 0.0263 | 0.0287 | 0.0373 | 0.0241 | 0.0256 | 0.0252 | 0.0249 | 0.0097 | 0.0103 | 0.0083 | 0.0018 | 0.0026 | 0.0029 | 0.0044 | 0.0011 | 0.0045 | 0.0026 | 0.0047 | 0.0029 |  |  |  |  |
| Beijing Han | 0.0023 | 0.0223 | 0.0493 | 0.0539 | 0.0255 | 0.0240 | 0.0287 | 0.0241 | 0.0257 | 0.0338 | 0.0251 | 0.0263 | 0.0261 | 0.0260 | 0.0088 | 0.0102 | 0.0081 | 0.0010 | 0.0023 | 0.0046 | 0.0054 | 0.0011 | 0.0067 | 0.0028 | 0.0032 | 0.0019 | 0.0014 |  |  |  |
| Henan Han | 0.0022 | 0.0228 | 0.0498 | 0.0543 | 0.0256 | 0.0246 | 0.0289 | 0.0247 | 0.0262 | 0.0344 | 0.0256 | 0.0264 | 0.0263 | 0.0262 | 0.0094 | 0.0105 | 0.0081 | 0.0014 | 0.0020 | 0.0035 | 0.0045 | 0.0004 | 0.0052 | 0.0023 | 0.0036 | 0.0021 | 0.0011 | 0.0006 |  |  |
| Shanghai Han | 0.0022 | 0.0243 | 0.0523 | 0.0568 | 0.0268 | 0.0255 | 0.0304 | 0.0256 | 0.0274 | 0.0355 | 0.0259 | 0.0272 | 0.0270 | 0.0268 | 0.0096 | 0.0108 | 0.0085 | 0.0015 | 0.0022 | 0.0030 | 0.0040 | 0.0004 | 0.0048 | 0.0019 | 0.0036 | 0.0021 | 0.0007 | 0.0006 | 0.0003 |  |
| Guangdong Han | 0.0032 | 0.0252 | 0.0547 | 0.0588 | 0.0282 | 0.0270 | 0.0319 | 0.0273 | 0.0290 | 0.0381 | 0.0258 | 0.0276 | 0.0268 | 0.0269 | 0.0100 | 0.0115 | 0.0100 | 0.0023 | 0.0032 | 0.0018 | 0.0030 | 0.0007 | 0.0034 | 0.0015 | 0.0050 | 0.0034 | 0.0009 | 0.0017 | 0.0010 | 0.0006 |
|  |  |  |  |  |  |  |  |  |  |  |  |  |  |  |  |  |  |  |  |  |  |  |  |  |  |  |  |  |  |  |
|  |  |  |  |  |  |  |  |  |  |  |  |  |  |  |  |  |  |  |  |  |  |  |  |  |  |  |  |  |  |  |
