# Supplementary material for: Population Genetic Diversity and Clustering Analysis for Chinese Dongxiang Group With 30 Autosomal InDel Loci Simultaneously Analyzed
Source: Front Genet. 2018 Aug 2;9:279. doi: 10.3389/fgene.2018.00279 (PMC6082941; doi:10.3389/fgene.2018.00279)
Supplement: TABLE S4 — Pairwise Fst values for Chinese Dongxiang group and the reference populations. [file Table_4.docx]

Supplemental Table 4. Pairwise *F_st_* values for Chinese Dongxiang group and the reference populations.

| Populations | Dongxiang | Cape Colored | Xhosa | Zulu | Chihuahua Mexican | Jalisco Mexican | Mexico Mexican | Veracruz Mexican | Yucatan Mexican | Amerindian Mexican | Dane | Hungarian | Basque | Central Spanish | Kazak | Uyghur | Hui | Xibe | Yi | Zhuang | Dong | Tujia | Miao | She | Tibet Tibetan | Qinghai Tibetan | Chengdu Han | Beijing Han | Henan Han | Shanghai Han |
| --- | --- | --- | --- | --- | --- | --- | --- | --- | --- | --- | --- | --- | --- | --- | --- | --- | --- | --- | --- | --- | --- | --- | --- | --- | --- | --- | --- | --- | --- | --- |
| Cape Colored | 0.0566 |  |  |  |  |  |  |  |  |  |  |  |  |  |  |  |  |  |  |  |  |  |  |  |  |  |  |  |  |  |
| Xhosa | 0.1453 | 0.0360 |  |  |  |  |  |  |  |  |  |  |  |  |  |  |  |  |  |  |  |  |  |  |  |  |  |  |  |  |
| Zulu | 0.1612 | 0.0479 | 0.0016 |  |  |  |  |  |  |  |  |  |  |  |  |  |  |  |  |  |  |  |  |  |  |  |  |  |  |  |
| Chihuahua Mexican | 0.0631 | 0.0294 | 0.0895 | 0.1119 |  |  |  |  |  |  |  |  |  |  |  |  |  |  |  |  |  |  |  |  |  |  |  |  |  |  |
| Jalisco Mexican | 0.0575 | 0.0244 | 0.0859 | 0.1094 | -0.0034 |  |  |  |  |  |  |  |  |  |  |  |  |  |  |  |  |  |  |  |  |  |  |  |  |  |
| Mexico Mexican | 0.0760 | 0.0278 | 0.0819 | 0.1070 | 0.0113 | 0.0058 |  |  |  |  |  |  |  |  |  |  |  |  |  |  |  |  |  |  |  |  |  |  |  |  |
| Veracruz Mexican | 0.0646 | 0.0343 | 0.0982 | 0.1172 | 0.0074 | 0.0012 | 0.0051 |  |  |  |  |  |  |  |  |  |  |  |  |  |  |  |  |  |  |  |  |  |  |  |
| Yucatan Mexican | 0.0713 | 0.0415 | 0.1055 | 0.1286 | 0.0103 | 0.0091 | 0.0085 | 0.0047 |  |  |  |  |  |  |  |  |  |  |  |  |  |  |  |  |  |  |  |  |  |  |
| Amerindian Mexican | 0.0981 | 0.0689 | 0.1202 | 0.1437 | 0.0232 | 0.0206 | 0.0234 | 0.0146 | 0.0128 |  |  |  |  |  |  |  |  |  |  |  |  |  |  |  |  |  |  |  |  |  |
| Dane | 0.0591 | 0.0428 | 0.1239 | 0.1397 | 0.0301 | 0.0267 | 0.0570 | 0.0423 | 0.0559 | 0.0926 |  |  |  |  |  |  |  |  |  |  |  |  |  |  |  |  |  |  |  |  |
| Hungarian | 0.0575 | 0.0353 | 0.1085 | 0.1265 | 0.0308 | 0.0287 | 0.0504 | 0.0443 | 0.0581 | 0.0962 | 0.0062 |  |  |  |  |  |  |  |  |  |  |  |  |  |  |  |  |  |  |  |
| Basque | 0.0620 | 0.0488 | 0.1287 | 0.1496 | 0.0370 | 0.0327 | 0.0609 | 0.0527 | 0.0686 | 0.1006 | 0.0116 | 0.0124 |  |  |  |  |  |  |  |  |  |  |  |  |  |  |  |  |  |  |
| Central Spanish | 0.0592 | 0.0299 | 0.0986 | 0.1179 | 0.0246 | 0.0213 | 0.0434 | 0.0394 | 0.0507 | 0.0829 | 0.0050 | 0.0041 | 0.0054 |  |  |  |  |  |  |  |  |  |  |  |  |  |  |  |  |  |
| Kazak | 0.0126 | 0.0359 | 0.1202 | 0.1370 | 0.0294 | 0.0240 | 0.0446 | 0.0308 | 0.0402 | 0.0690 | 0.0294 | 0.0286 | 0.0368 | 0.0271 |  |  |  |  |  |  |  |  |  |  |  |  |  |  |  |  |
| Uyghur | 0.0132 | 0.0324 | 0.1170 | 0.1357 | 0.0284 | 0.0252 | 0.0450 | 0.0354 | 0.0433 | 0.0731 | 0.0234 | 0.0185 | 0.0297 | 0.0194 | 0.0028 |  |  |  |  |  |  |  |  |  |  |  |  |  |  |  |
| Hui | 0.0242 | 0.0639 | 0.1402 | 0.1541 | 0.0777 | 0.0749 | 0.0851 | 0.0718 | 0.0746 | 0.0980 | 0.0784 | 0.0752 | 0.0841 | 0.0719 | 0.0353 | 0.0341 |  |  |  |  |  |  |  |  |  |  |  |  |  |  |
| Xibe | 0.0065 | 0.0741 | 0.1633 | 0.1761 | 0.0794 | 0.0743 | 0.0910 | 0.0744 | 0.0827 | 0.1093 | 0.0755 | 0.0784 | 0.0817 | 0.0768 | 0.0193 | 0.0267 | 0.0287 |  |  |  |  |  |  |  |  |  |  |  |  |  |
| Yi | 0.0078 | 0.0674 | 0.1582 | 0.1710 | 0.0789 | 0.0740 | 0.0900 | 0.0748 | 0.0799 | 0.1119 | 0.0776 | 0.0801 | 0.0868 | 0.0791 | 0.0260 | 0.0311 | 0.0262 | 0.0019 |  |  |  |  |  |  |  |  |  |  |  |  |
| Zhuang | 0.0121 | 0.0798 | 0.1799 | 0.1959 | 0.0858 | 0.0818 | 0.0988 | 0.0861 | 0.0916 | 0.1237 | 0.0740 | 0.0786 | 0.0751 | 0.0737 | 0.0264 | 0.0315 | 0.0329 | 0.0085 | 0.0102 |  |  |  |  |  |  |  |  |  |  |  |
| Dong | 0.0217 | 0.1065 | 0.2087 | 0.2240 | 0.1161 | 0.1078 | 0.1288 | 0.1094 | 0.1170 | 0.1474 | 0.0999 | 0.1047 | 0.1083 | 0.1069 | 0.0442 | 0.0510 | 0.0461 | 0.0155 | 0.0172 | 0.0057 |  |  |  |  |  |  |  |  |  |  |
| Tujia | 0.0072 | 0.0857 | 0.1782 | 0.1917 | 0.0925 | 0.0868 | 0.1023 | 0.0872 | 0.0940 | 0.1193 | 0.0885 | 0.0913 | 0.0942 | 0.0912 | 0.0299 | 0.0344 | 0.0321 | 0.0032 | 0.0034 | 0.0034 | 0.0055 |  |  |  |  |  |  |  |  |  |
| Miao | 0.0178 | 0.0910 | 0.1985 | 0.2126 | 0.1026 | 0.0960 | 0.1128 | 0.0998 | 0.1035 | 0.1406 | 0.0900 | 0.0923 | 0.1037 | 0.0972 | 0.0381 | 0.0430 | 0.0385 | 0.0209 | 0.0139 | 0.0070 | 0.0055 | 0.0112 |  |  |  |  |  |  |  |  |
| She | 0.0130 | 0.0896 | 0.1901 | 0.2027 | 0.0943 | 0.0898 | 0.1049 | 0.0899 | 0.0977 | 0.1221 | 0.0904 | 0.0946 | 0.0994 | 0.0963 | 0.0349 | 0.0398 | 0.0385 | 0.0091 | 0.0078 | 0.0076 | 0.0063 | 0.0035 | 0.0088 |  |  |  |  |  |  |  |
| Tibet Tibetan | 0.0068 | 0.0684 | 0.1554 | 0.1741 | 0.0702 | 0.0634 | 0.0800 | 0.0682 | 0.0691 | 0.0935 | 0.0781 | 0.0757 | 0.0863 | 0.0767 | 0.0184 | 0.0239 | 0.0203 | 0.0102 | 0.0112 | 0.0170 | 0.0257 | 0.0108 | 0.0234 | 0.0178 |  |  |  |  |  |  |
| Qinghai Tibetan | 0.0043 | 0.0631 | 0.1474 | 0.1630 | 0.0725 | 0.0667 | 0.0814 | 0.0697 | 0.0740 | 0.1007 | 0.0766 | 0.0750 | 0.0856 | 0.0773 | 0.0199 | 0.0252 | 0.0192 | 0.0044 | 0.0035 | 0.0121 | 0.0181 | 0.0059 | 0.0170 | 0.0111 | 0.0010 |  |  |  |  |  |
| Chengdu Han | 0.0053 | 0.0788 | 0.1741 | 0.1873 | 0.0886 | 0.0838 | 0.1026 | 0.0869 | 0.0964 | 0.1228 | 0.0787 | 0.0816 | 0.0831 | 0.0808 | 0.0279 | 0.0291 | 0.0307 | 0.0045 | 0.0036 | 0.0038 | 0.0090 | 0.0019 | 0.0108 | 0.0059 | 0.0151 | 0.0081 |  |  |  |  |
| Beijing Han | 0.0055 | 0.0769 | 0.1660 | 0.1794 | 0.0863 | 0.0805 | 0.0958 | 0.0807 | 0.0878 | 0.1125 | 0.0847 | 0.0863 | 0.0887 | 0.0868 | 0.0256 | 0.0306 | 0.0305 | 0.0016 | 0.0026 | 0.0106 | 0.0126 | 0.0018 | 0.0207 | 0.0068 | 0.0095 | 0.0044 | 0.0028 |  |  |  |
| Henan Han | 0.0054 | 0.0794 | 0.1678 | 0.1808 | 0.0874 | 0.0832 | 0.0972 | 0.0834 | 0.0902 | 0.1152 | 0.0877 | 0.0879 | 0.0903 | 0.0887 | 0.0284 | 0.0321 | 0.0308 | 0.0036 | 0.0022 | 0.0073 | 0.0107 | -0.0002 | 0.0152 | 0.0058 | 0.0116 | 0.0057 | 0.0019 | 0.0006 |  |  |
| Shanghai Han | 0.0059 | 0.0859 | 0.1750 | 0.1872 | 0.0924 | 0.0872 | 0.1029 | 0.0876 | 0.0950 | 0.1188 | 0.0897 | 0.0927 | 0.0937 | 0.0921 | 0.0296 | 0.0336 | 0.0327 | 0.0045 | 0.0040 | 0.0059 | 0.0087 | 0.0002 | 0.0137 | 0.0043 | 0.0119 | 0.0061 | 0.0007 | 0.0006 | 0.0002 |  |
| Guangdong Han | 0.0095 | 0.0885 | 0.1828 | 0.1946 | 0.0973 | 0.0926 | 0.1081 | 0.0935 | 0.1006 | 0.1283 | 0.0889 | 0.0925 | 0.0930 | 0.0919 | 0.0307 | 0.0359 | 0.0373 | 0.0073 | 0.0076 | 0.0012 | 0.0047 | 0.0010 | 0.0081 | 0.0032 | 0.0168 | 0.0105 | 0.0012 | 0.0046 | 0.0027 | 0.0009 |
|  |  |  |  |  |  |  |  |  |  |  |  |  |  |  |  |  |  |  |  |  |  |  |  |  |  |  |  |  |  |  |
